# Novel Benzoxazoles Containing 4-Amino-Butanamide Moiety Inhibited LPS-Induced Inflammation by Modulating IL-6 or IL-1β mRNA Expression

**DOI:** 10.3390/ijms23105331

**Published:** 2022-05-10

**Authors:** Jihye Yoo, Jiyoung Park, Darong Kim, Yeonjoo Huh, Hea-Young Park Choo, Hyun Ae Woo

**Affiliations:** 1 Graduate School of Pharmaceutical Sciences, College of Pharmacy, Ewha Womans University, Seoul 03760, Korea; jibong9077@ewhain.net (J.Y.); jypark89@ewhain.net (J.P.); kdrlj237@ewhain.net (D.K.); godq07@ewhain.net (Y.H.); hypark@ewha.ac.kr (H.-Y.P.C.); 2 Fluorescence Core Imaging Center, Department of Life Science, Ewha Womans University, Seoul 03760, Korea

**Keywords:** benzoxazole, small molecules, IL-1β, IL-6, TNF-α, mRNA expression, anti-inflammatory effect

## Abstract

LPS induces inflammatory cytokines, including IL-1β, IL-6, and TNF-α, and causes an inflammatory response. The development of small molecules that have suppressive effect on those inflammatory cytokines is a desirable strategy for the treatment of inflammatory diseases. We synthesized 12 novel compounds with 4-amino-*N*-(4-(benzo[*d*]oxazol-2-ylamino)phenyl)butanamide moiety and evaluated their biological activities. Among them, 4 compounds (compound **5d, 5c, 5f, 5m** and synthetic intermediate **4d**) showed potent inhibition activities on IL-1β and IL-6 mRNA expression in vitro. Further, in vivo activity was evaluated with two compounds (**5f** and **4d**) and mRNA levels of IL-1β, IL-6, and TNF-α were significantly decreased without hepatotoxicity. From the in vivo and in vitro test results, we confirmed that our synthesized compounds are effective for suppression of representative inflammatory cytokines.

## 1. Introduction

Inflammation is an essential immune response of the host to the exposure of tissues and organs to harmful stimuli such as microbial pathogens, irritants, or toxic cellular components that cause the ultimate restoration of tissue structure and function [1]. Innate and adaptive immune response are two main integral components of the host’s defense system which are critical for pathogen-specific defense and immunological memory [2]. In the innate immune system, many classes of pathogens are recognized by their common molecular patterns. There are various types of pathogen-associated molecular patterns (PAMP), including lipopolysaccharides (LPS), aldehyde-derivatized proteins, mannans, teichoic acids, denatured DNA, and bacterial DNA. Pathogen-recognition receptors (PRR) are used for recognition of PAMP by a group of germline coded and evolutionary conserved proteins [3].

Lipopolysaccharide (LPS) is the major component of Gram-negative bacteria cell walls [4]. As it stimulates the release of inflammatory cytokines in various cell types, bacterial LPS has been extensively used to establish an inflammatory model [5]. LPS is initially extracted from bacterial membranes by LPS binding protein (LPB) in serum. LPS is transferred to CD14 by LBP, cleaved into monomeric molecules, and presented to the TLR4-MD-2 complex. Binding and aggregation of the TLR4-MD-2 complex with LPS leads to the activation of multiple signaling components such as NK-kB and IRF3. Subsequently, various pro-inflammatory cytokines are produced and inflammatory response occurs [6].

An inflammatory response is induced by LPS secreting inflammatory cytokines. Representative inflammatory cytokines include IL-1b, IL-6, and TNF-α [7]. IL-1β has a stimulatory effect on CD4+ T cells and differentiation into the T helper cells [8]. IL-1β is synthesized by various cell types such as monocytes, macrophages, neutrophils, hepatocytes, and tissue macrophages [9]. IL-6 is involved in hematopoiesis and final maturation of B-cells into antibody-producing plasma cells, T cell activation, and differentiation. IL-6 is expressed by mononuclear phagocytes, T cells, B cells, fibroblasts, endothelial cells, keratinocytes, hepatocytes, and bone marrow cells [10]. TNF-α is involved in stimulation of the proliferation of normal cells, exerts cytolytic or cytostatic activity against tumor cells, and induces inflammatory, antiviral, and immunoregulatory effects [11]. TNF-α is primarily secreted from activated macrophages [12].

**Figure 1:**
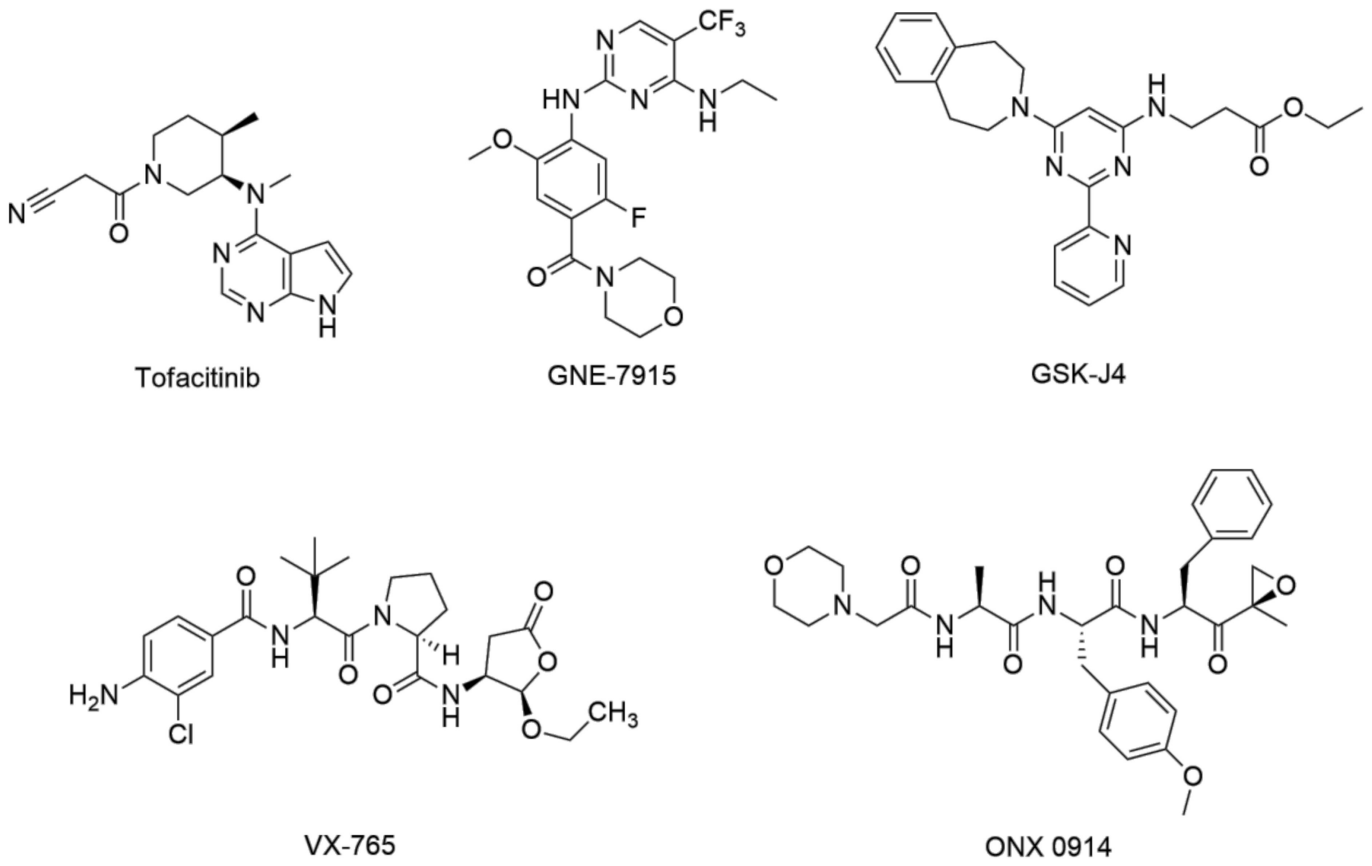
Chemical structures of small molecule inflammatory cytokine modulators.

A number of small molecules have been developed to treat immune disorders by regulating cytokine function. They act on proteins involved in the cytokine production pathway to regulate cytokine production and signaling. So far, tofacitinib, GNE-7915, GSK-J4, VX-765, and ONX 0914 have been developed as representative inflammatory cytokine inhibitors (Figure 1) [13,14,15,16,17,18,19,20,21,22,23,24]. Unlike biopharmaceuticals such as protein-based therapies, these small molecule modulators can act on intracellular proteins and modulate abnormal cytokine signaling or downstream sequences.

In our previous studies, we synthesized various benzoxazole derivatives and investigated their biological activities, particularly their anti-inflammatory effect [25,26,27,28,29]. We developed the compounds with 5-LOX inhibition effect. With an improvement effect of methacholine-induced airway hypersensitivity, it was confirmed that it has potential as an asthma treatment [27]. In addition, IL-6-mediated STAT3 phosphorylation inhibitors were developed. The compounds also showed the inhibitory effect on the secretion of inflammatory cytokines from effector Th1, Th2, and Th17 cells. From the results, we confirmed that the compounds have the potential to be candidates for rheumatoid arthritis (RA) treatment [28]. Further, we developed the compounds that have an inhibitory effect on IL-1β, IL-6, IL-13, TNF-α, perilipin (PLIN) 2, and PLIN 3 expression from bone marrow-derived mast cells (BMMC) activated by LPS [29]. The *N*-phenylbenzo[*d*]oxazol-2-amine derivatives synthesized in previous studies have amino, methyl, or chloro substituents at the 5th position (R_2_). In addition, relatively small substituents were present on the phenyl ring connected to the benzoxazole by an amino linker, such as 4-ethyl, 3-bromo or 2-methoxy, and 4-nitro groups.

Meanwhile, the compound synthesized in this study may have methyl substituent (R_1_) at the 4th position (R_1_), methyl, fluoro, *t*-butyl, methoxy or chloro substituent at the 5th position (R_2_), or methyl substituent on the 6th position (R_3_). Further, methoxy substituent may be present at the 2-position of the phenyl group (R_4_) linked to the benzoxazole by amino linker. In particular, the 4-amino-butanamide substituent is characterized by a longer alkyl amino chain and is in the 4th position of the phenyl group (R_5_).

This study synthesized benzoxazole derivatives with 4-amino-butanamide moiety and investigated their LPS-induced inflammatory response inhibitory effects. We investigated if and how benzoxazole derivatives can have an inhibitory effect on LPS-induced inflammation and whether it has the potential to be useful in the treatment of inflammation diseases by inflammation cytokines. We employed two types of cells and a mouse model of LPS-induced inflammation to examine whether the benzoxazole derivatives can have excellent suppressive effects in LPS-induced acute inflammation and how they can be modulated.

## 2. Results

### 2.1. Synthesis of 4-Amino-N-(4-(benzo[D]oxazol-2-ylamino)phenyl)butanamide Derivatives

**Scheme 1:**
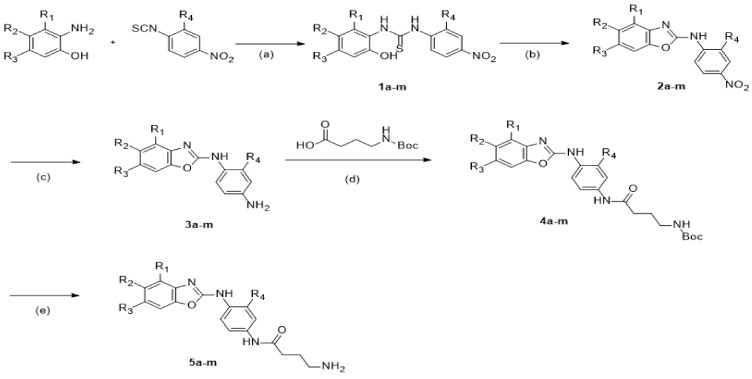
Synthesis of 4-amino-*N*-(4-(benzo[*d*]oxazol-2-ylamino)phenyl)butanamide derivatives. (**a**) MeOH, rt, 24 hrs, (**b**) KO_2_, acetonitrile, N_2_, rt, 16 hrs, (**c**) Pd/C, H_2_, rt, 30 min, (**d**) *N*-boc-γ-aminobutyric acid, PyBop, diisopropylethylamine, rt, 16 hrs, (**e**) 4 M HCl in dioxane, DCM, rt, 3 hrs.

Novel compounds with 4-amino-*N*-(4-(benzo[*d*]oxazol-2-ylamino)phenyl)butanamide derivatives were synthesized as shown in Scheme 1. As the starting materials, variously substituted 2-aminophenol were commercially obtained. 1-Isothiocyanato-4-nitrobenzene or 1-isothiocyanato-2-methoxy-4-nitrobenzene were reacted with them to give thiourea compounds (compounds **1a**–**m**). The thiourea compounds were cyclized with KO_2_ to obtain benzoxazole compounds by oxidation process (compounds **2a**–**m**). The nitro groups were reduced to amino groups with Pd/C under H_2_ atmosphere (compounds **3a**–**m**). The resulting amino groups were reacted with *N*-*tert*-butyloxycarbonyl (boc)-γ-aminobutyric acid (compounds **4a**–**m**) and *N*-boc groups were removed with 4 M HCl in dioxane to obtain 4-amino-*N*-(4-(benzo[*d*]oxazol-2-ylamino)phenyl)butanamide derivatives (compounds **5a**–**m**).

### 2.2. In Vitro Inhibition of mRNA Expression Levels of IL-6 and IL-1β

We screened 13 novel synthetic compounds (compounds **5a**–**m**) and three of their intermediates (compounds **4d**, **4e**, and **4l**) by measuring mRNA expression levels of IL-6 and IL-1β. Human keratinocyte HaCaT cell was used for the evaluation of suppressive effect of mRNA expression. Compounds **5d**, **5c**, **5f**, **5m**, and synthetic intermediate **4d** showed the potent activities (Table 1). Compounds were administered at a concentration of 10 μM. IL-6 mRNA expression was 5.3 for compound **4d**, 4.6 for compound **5c**, 7.5 for compound **5d**, 7.2 for compound **5f**, and 9.0 for compound **5m**. IL-6 mRNA expression was significantly lower than when inflammation was induced by administration of LPS alone.

Dose-response study was conducted with the 5 compounds at different concentrations to obtain IC_50_ (uM) (Table 2). IC_50_ (uM) of compound **4d** was 6.04 × 10^−5^, compound **5c** was 1.64, compound **5d** was 3.27 × 10^−2^, compound **5f** was 4.44 × 10^−4^, and compound **5m** was 1.99 × 10^−4^. Cell viability was measured by MTT assay, and the compounds did not show cytotoxicity (data now shown).

**Figure 2:**
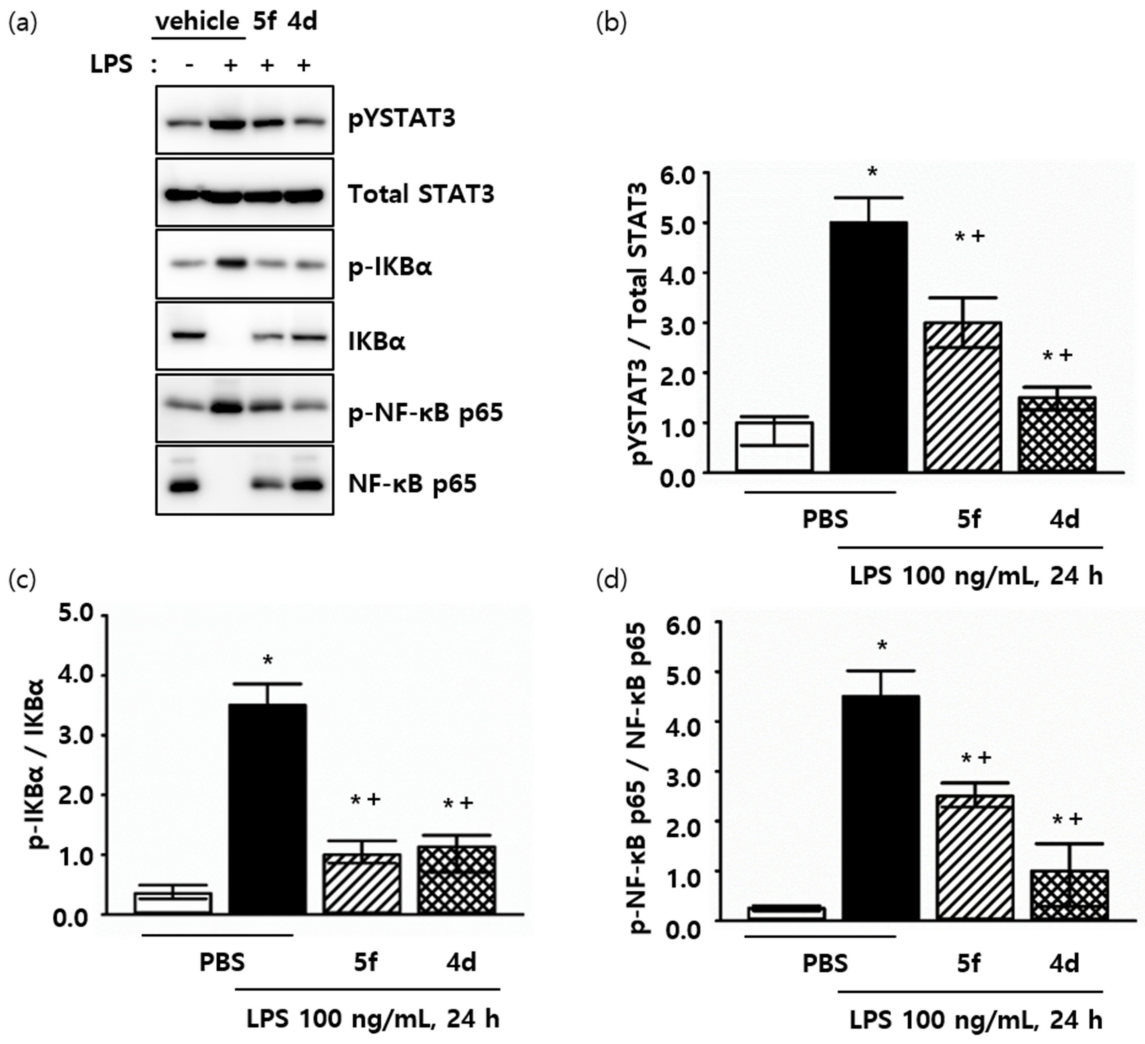
Compounds **5f** and **4d** attenuate inflammation in vitro. Effects of compounds (10 µM) on the protein levels of STAT3, IκB, and NF-κB in AML-12 cells. (**a**) Expression levels of inflammation related-proteins in vitro. (**b**–**d**) Protein expression levels were normal-ized against the indicated protein. * *p* < 0.05 compared with the group treated with the PBS (vehicle). + *p* < 0.05 compared with the group treated with the LPS. Data are pre-sented as the mean ± SD.

To assess the inhibitory effects of two compounds (**4d** and **5f**) on the LPS-induced signaling of inflammation in human liver hepatocytes AML-12 cells, we tested their inhibition effects on the hepatocytes using a concentration range, where no cytotoxicity was observed. We performed a Western blotting assay to confirm changes in STAT3 or NF-κB activity and found that the phosphorylation of STAT3 or IκB-α was prevented in AML-12 cells treated with two compounds, and statistical significance was confirmed in the group treated with 10 μM compounds. As shown in Figure 2, the expression levels of pY-STAT3, p-IκBα, and p-NF-κB p65 in AML-12 cells were significantly downregulated by compounds **5f** and **4f**. Many studies have shown that LPS causes the inflammatory response by activating the STAT3 or NF-κB pathway and we expected that compounds of **4d** and **5f** modulate inflammation by inhibiting STAT3/NF-κB activity.

### 2.3. In Vivo Inhibition of mRNA Expression Levels of IL-6 and IL-1β in Mice

**Figure 3:**
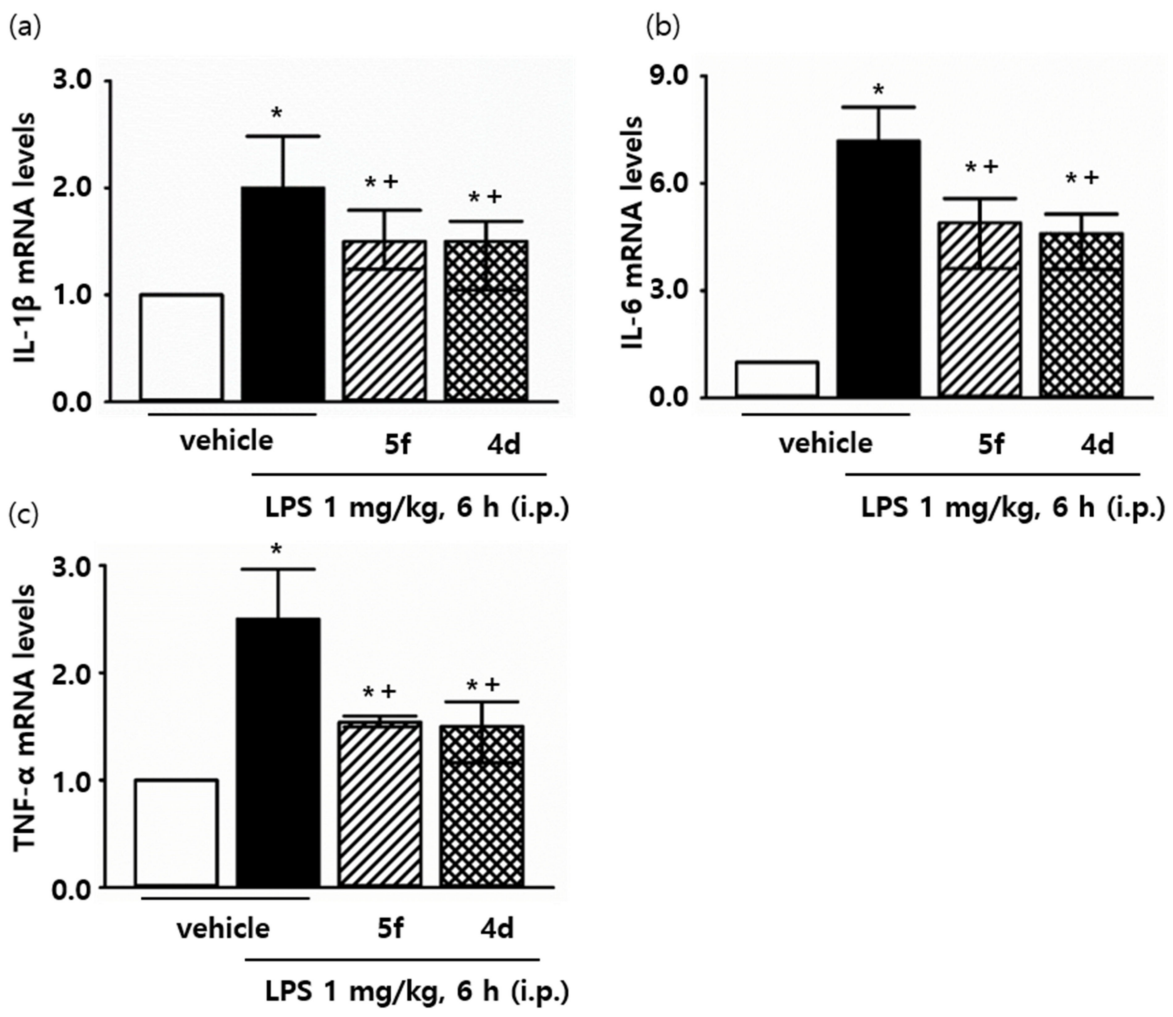
mRNA expression levels of inflammatory cytokines after treatment of compounds **5f** and **4d**. (**a**) mRNA expression level of IL-1β, (**b**) mRNA expression level of IL-6, (**c**) mRNA expression level of TNF-α. * *p* < 0.05 compared with the group treated with the PBS (vehicle). + *p* < 0.05 compared with the group treated with the LPS. Data are presented as the mean ± SD.

Two compounds (**4d** and **5f**) were selected for the in vivo assay. Compounds were diluted with olive oil and injected into mice 1 h before LPS (1 mg/kg, 6 h) was administered to the mice. LPS was resuspended in saline solution and injected i.p. at a dose of 1 mg/kg of body weight. At the end of the trial, mice were sacrificed, and blood and liver were collected. For the evaluation of anti-inflammatory activity in mice, mRNA expression of IL-1β, IL-6, and TNF-α were measured. The results are shown in Figure 3.

### 2.4. Liver Morphology and Macrophage Infiltration

Liver histology of LPS treatment showed inflammatory activity compared to the control (Figure 3a). As a result of LPS administration, macrophage infiltration was observed in liver tissue. When compounds **4f** and **5d** were administered, it was observed that the inflammatory response was improved compared to when LPS alone was administered.

**Figure 4:**
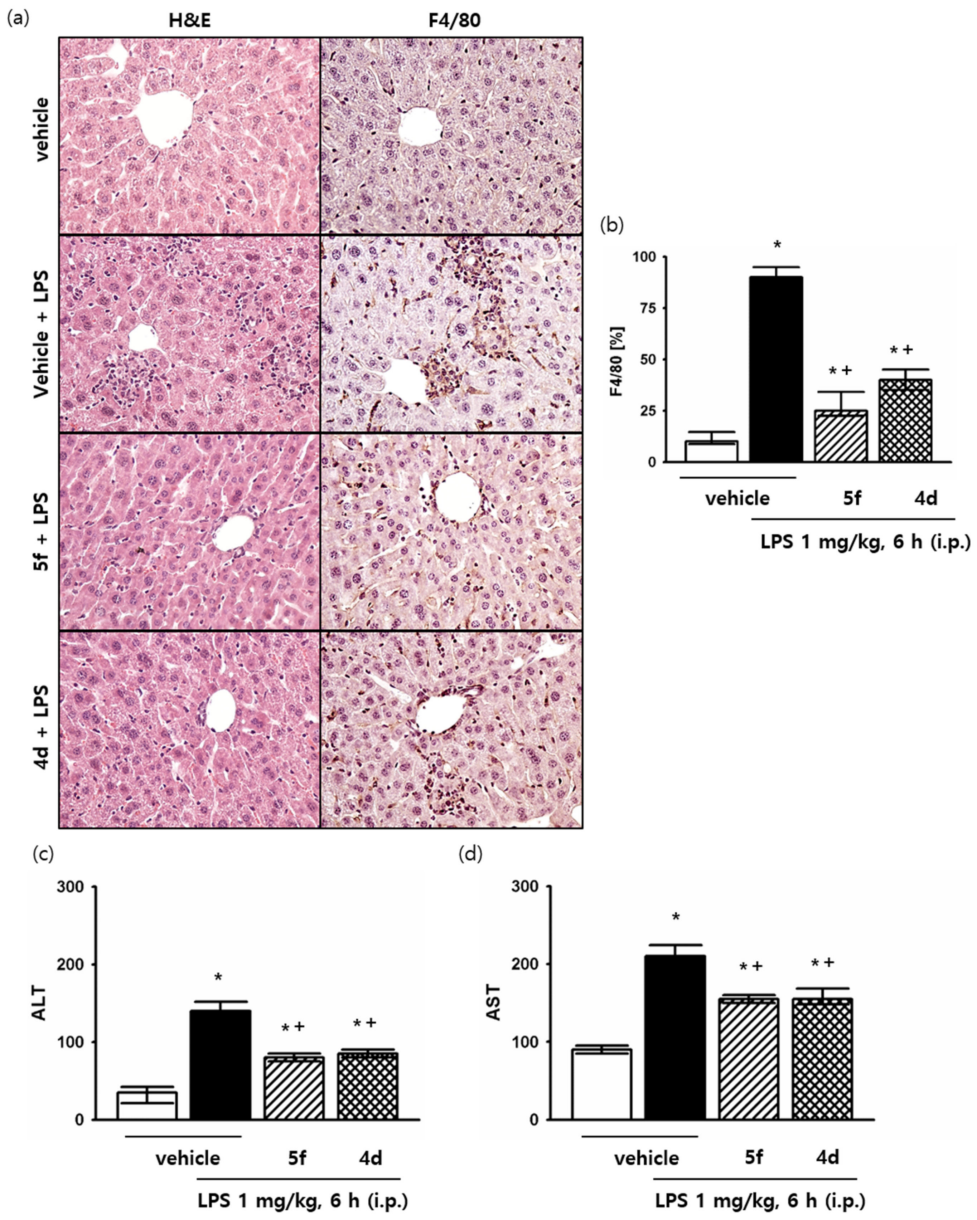
Protective effects of compounds **5f** and **4d** in vivo models for LPS induced-liver in-flammation disease. (**a**,**b**) Histopathological examination was performed using H&E staining and immunohistochemical staining using F4/80 antibody, the F4/80 positive areas were quantified, magnification are ×400. (**c**,**d**) Effects of compounds on serum ALT and AST levels during liver inflammation. * *p* < 0.05 compared with the group treated with the PBS (vehicle). + *p* < 0.05 compared with the group treated with the LPS. Data are presented as the mean ± SD.

In order to characterize the phenotype of macrophages that accumulated in the LPS-induced inflammation, an immunohistochemical study was performed using antibodies against F4/80, a marker of mature macrophages (Figure 4b). When LPS alone was administered to induce inflammatory response, hepatic macrophage accumulation increased. As a result, there was a marked elevation of the number of F4/80-positive cells. When compounds **4f** and **5d** were administered, a significant decrease of F4/80 positive cells was observed.

In accordance with altered histological features, plasma alanine transferase (ALT) and aspartate transaminase (AST) levels were increased after LPS administration (Figure 4c,d), and when compounds **4f** and **5d** were administered, ALT and AST levels decreased.

## 3. Discussion

Here in this study, we synthesized novel compounds with benzoxazole moieties and investigated their suppressive effect on the mRNA expression of inflammatory cytokines and in vivo anti-inflammatory effect on LPS administration. A total of 13 compounds were synthesized by linking tert-butyloxycarbonyl (boc)-γ-butyric acid to the benzoxazole moieties with various substituents, followed by the removal of boc protection group by hydrolysis under acidic condition. Biological activity evaluation was carried out on 13 final compounds and three intermediates before boc protecting group hydrolysis.

To induce inflammatory response, lipopolysaccharide (LPS) was used for in vitro and in vivo tests. LPS is the bacterial cell wall of Gram-negative bacteria and is generally recognized as a potent activator of monocytes/macrophages, and its effects include an altered production of key mediators, such as inflammatory cytokines. LPS is also known as pro-skin inflammation agent and many studies used LPS as the stimuli to construct in vitro cell skin inflammation model [30,31].

Bacterial LPS has been widely used to study inflammation due to the abundance of inflammatory effects that it generates through TLR signaling. TLRs are transmembrane receptors with an extracellular domain which interacts with a pathogen ligand and an intracellular domain involved in signaling. Especially, TLRs play an important role in the recognition of pathogens in keratinocytes. In this study, we used LPS to induce inflammation in human keratinocyte HaCat cells and investigated the inhibitory effects of the compounds on inflammatory cytokine expression [32,33].

We examined mRNA expression levels of IL-6 and IL-1β of the experimental group and compared with those of the control groups. The inhibitory effects of the compounds on IL-6 and IL-1β showed in similar pattern. Compounds that were most effective in inhibiting IL-6 mRNA expression (compounds **5c**, **5d**, **5f**, **5m**, and **4d**) were also the most effective in inhibiting IL-1β mRNA expression. Except for compound **5m**, the compounds with potent activity were compounds in which the 2-position of the benzene ring (R_4_) was not substituted with a methoxy group. Compounds **5f** and **4d**, which showed high activity in both IL-6 and IL-1β, were substituted with a *tert*-butyl group and a methyl group at the 5th position of benzoxazole (R_2_), respectively. Compound **4d**, an intermediate without removing the boc protecting group, showed high anti-inflammatory activity compared to other intermediates (compounds **4e** and **4l**). Among the compounds, when the substituent at the R_2_ position was a *tert*-butyl group, the activity was good regardless of the presence or absence of a methoxy group at the R_4_ position (compounds **5f** and **5m**). In the case of a methyl group at the R_2_ position, the activity was generally good regardless of the presence or absence of a methoxy group at the R_4_ position and a boc group at the R_5_ position. When there was a methyl group at the R_3_ position, the activity was not very good.

From the in vitro tests, two compounds were selected and were evaluated in vivo tests to test inhibition activity on the expression of IL-6, IL-1β, and TNF-α by injecting LPS into mice. Following to the LPS administration, severe macrophage infiltration was observed in liver tissue while administration of compounds **5f** and **4d** alleviated the inflammatory response. We also checked mRNA levels of IL-6, IL-1β, and TNF-α levels in the liver tissue. Compounds **5f** and **4d** showed significant suppressive activity on IL-6, IL-1β, and TNF-α expression levels. In addition, the alanine aminotransferase (ALT) and aspartate aminotransferase (AST) levels were examined to investigate the hepatotoxicity of the compounds, which was confirmed to be lower than the control group injected LPS only. We confirmed that the potent anti-inflammatory activity of the two compounds through in vivo study, and it was found that the difference in activity between the two compounds was not significant.

Through LPS-induced inflammatory responses, proinflammatory cytokines IL-6, IL-1β, and TNF-α are activated. It is important to effectively control the cytokines since the cytokines play an important role in the inflammatory response.

In this study, we developed novel benzoxazole derivatives and confirmed their anti-inflammatory activity by modulating pro-inflammatory cytokine expressions. From the in vivo and in vitro test results, we confirmed that our synthesized compounds are effective for suppression of representative inflammatory cytokines such as IL-1β, IL-6 and TNF-α. Therefore, it is possible to develop small molecule modulators for inflammatory diseases from these compounds through further study in the future.

## 4. Materials and Methods

### 4.1. Materials and Methods

Melting points were measured on an electro thermal digital melting point (Büchi, Germany) without calibration. ^1^H-NMR spectra were recorded on Varian NMR AS and Varian Unity Inova 400 MHz NMR spectrometers. Chemical shifts were reported in parts per million (*δ*) units relative to the solvent peak. The ^1^H NMR data were reported as peak multiplicities: s for singlet; d for doublet; t for triplet; q for quartet; and m for multiplet. Coupling constants were recorded in hertz (Hz). MS spectra were measured using Jeol JMS 700 high resolution mass spectrometer from the Korea Basic Science Institute (Daegu). Reagents were of commercial grade and were purchased from Sigma-Aldrich Co. (St. Louis, MI, USA), Merck (Darmstadt, Geramny), Duksan Pure Chemical Co (Ansan, Korea).

### 4.2. Synthesis of 1-(Substituted-2-hydroxyphenyl)-3-(4-nitrophenyl)thioureas (***1a**–**g***)

First, 25 mL of methanol was added to variously substituted 2-amino-4-nitrophenol (100 mg, 1 eq.) and 1-isothiocyanato-4-nitrobenzene or 1-isothiocyanato-2-methoxy-4-nitrobenzene (1 eq.). The reaction mixture was stirred at room temperature for 24 h. After reaction, the organic solvent was removed under reduced pressure to obtain 1-(substituted-2-hydroxyphenyl)-3-(4-nitrophenyl)thioureas or 1-(2-hydroxyphenyl)-3-(2-methoxy-4-nitrophenyl)thioureas (**1a**–**m**).

1-(2-Hydroxyphenyl)-3-(4-nitrophenyl)thiourea (**1a**)

Yellow solid (95.9%), mp 148–150 °C, ^1^H-NMR (DMSO-*d*_6_, 400 MHz) *δ* 8.30 (dd, *J* = 7.2, 2.0 Hz, 1 H), 8.25 (dd, *J* = 7.2, 2.0 Hz, 1 H), 7.90 (brs, 1 H), 7.83 (dd, *J* = 7.2, 2.0 Hz, 1 H), 7.70 (d, *J* = 9.2 Hz, 1 H), 7.30 (d, *J* = 7.2 Hz, 1 H), 7.09 (dd, *J* = 9.2, 1.2 Hz, 1 H), 7.03 (td, *J* = 7.9, 1.2 Hz, 1 H), HR-FABMS Calcd for C_13_ H_12_N_3_O_3_S (M^+^ + H): 290.0601, Found: 290.0592.

1-(5-Fluoro-2-hydroxyphenyl)-3-(4-nitrophenyl)thiourea (**1b**)

Greenish brown solid (78.0%), mp 139–141 °C, ^1^H-NMR (DMSO-*d*_6_), 400 MHz, *δ* 10.71 (s, 1 H), 10.04 (s, 1 H), 9.59 (s, 1 H), 8.22 (dt, *J* = 9.2, 2.4 Hz, 2 H), 7.98–7.94 (m, 3 H) 6.89–6.85 (m, 2 H), HR-FABMS Calcd for C_13_H_11_FN_3_O_3_S (M^+^ + H): 308.0500, Found: 308.0503.

1-(2-Hydroxy-6-methylphenyl)-3-(4-nitrophenyl)thiourea (**1c**)

Yellow solid (91.3%), mp 168–170 °C, ^1^H-NMR (CDCl_3_, 400 MHz) *δ* 8.23 (dd, *J* = 9.0, 2.8 Hz, 2 H), 7.83 (dd, *J* = 7.2, 2.4 Hz, 2 H), 7.16 (t, *J* = 3.2, 2.4 Hz, 2 H), 6.94 (t, *J* = 8.0, 7.2 Hz, 1 H), HR-FABMS Calcd for C_14_H_14_N_3_O_3_S (M^+^ + H): 304.3443, Found: 304.0751.

1-(2-Hydroxy-5-methylphenyl)-3-(4-nitrophenyl)thiourea (**1d**)

Yellow solid (85.8%), mp 158–159 °C, ^1^H-NMR (CDCl_3_, 400 MHz) *δ* 8.25 (dt, *J* = 9.4, 2.6 Hz, 2 H), 7.86 (brs, 1 H), 7.73 (brs, 1 H), 7.69 (d, *J* = 9.2 Hz, 2 H), 7.11 (dd, *J* = 8.2, 1.8 Hz, 1 H), 7.07 (s, 1 H), 6.98 (d, *J* = 8.4 Hz, 1 H), 2.31 (s, 3 H), HR-FABMS Calcd for C_14_H_14_N_3_O_3_S (M^+^ + H): 304.3443, Found: 304.0752.

1-(2-Hydroxy-4-methylphenyl)-3-(4-nitrophenyl)thiourea (**1e**)

Mustard solid (91.3%), mp 130–136 °C, ^1^H-NMR (CDCl_3_, 400 MHz) *δ* 8.23 (dd, *J* = 7.2, 2.0 Hz, 2 H), 7.83 (brs, 1 H), 7.71 (d, *J* = 9.2 Hz, 2 H), 7.15 (d, *J* = 8.0 Hz, 1 H), 6.90 (s, 1 H), 6.83 (d, *J* = 8.0 Hz, 1 H), 2.36 (s, 3 H), HR-FABMS Calcd for C_14_H_14_N_3_O_3_S (M^+^ + H): 304.3443, Found: 304.0749.

1-(5-(*tert*-Butyl)-2-hydroxyphenyl)-3-(4-nitrophenyl)thiourea (**1f**)

Greyish yellow solid (71.8%), mp 141–145 °C, ^1^H-NMR (CDCl_3_, 400 MHz) *δ* 8.25 (d, *J* = 8.8 Hz, 2 H), 7.92 (brs, 1 H), 7.80 (brs, 1 H), 7.70 (d, *J* = 9.2 Hz, 2 H), 7.33 (dd, *J* = 8.8, 2.4 Hz, 1 H), 7.25 (d, *J* = 2.4 Hz, 1 H), 7.02 (d, *J* = 8.4 Hz, 1 H), 1.3 (s, 9 H), HR-FABMS Calcd for C_17_H_20_N_3_O_3_S (M^+^ + H): 346.4240, Found: 346.1221.

1-(2-Hydroxy-5-methoxyphenyl)-3-(4-nitrophenyl)thiourea (**1g**)

Green powder (89.0%), mp 122–124 °C, ^1^H-NMR (CDCl_3_, 400 MHz) *δ* 8.30–8.22 (m, 3 H), 7.85 (s, 1 H), 7.74 (s, 1 H), 7.67 (d, *J* = 7.2 Hz, 2 H), 7.03–7.00 (m, 1 H), 6.88–6.86 (m, 2 H), 3.78 (s, 3 H), HR-FABMS Calcd for C_14_H_14_N_3_O_4_S (M^+^ + H): 320.07, Found: 320.0699.

### 4.3. Synthesis of 1-(Substituted-2-hydroxyphenyl)-3-(2-methoxy-4-nitrophenyl)thioureas (***1h**–**m***)

Same procedure as 4.2. Synthesis of 1-(substituted-2-hydroxyphenyl)-3-(4-nitrophenyl)thioureas with 1-isothiocyanato-2-methoxy-4-nitrobenzene (1 eq.).

1-(2-Hydroxyphenyl)-3-(2-methoxy-4-nitrophenyl)thiourea (**1h**)

Light yellow solid (90.6%), mp 135–138 °C, ^1^H-NMR (CDCl_3_, 400 MHz) *δ* 8.85 (d, *J* = 8.8 Hz, 1 H), 8.37 (s, 1 H), 7.92 (dd, *J* = 8.8, 2.4 Hz, 1 H), 7.71 (d, *J* = 2.4 Hz, 1 H), 7.32 (q, *J* = 8.0 Hz,1 H), 7.09 (d, *J* = 7.2 Hz, 1 H), 7.04 (td, *J* = 7.7, 1.0 Hz, 1 H), 6.04 (brs, 1 H), 3.84 (s, 3 H), HR-FABMS Calcd for C_14_H_14_N_3_O_4_S (M^+^ + H): 320.3437, Found: 320.0700.

1-(5-Fluoro-2-hydroxyphenyl)-3-(2-methoxy-4-nitrophenyl)thiourea (**1i**)

Yellow solid (99.3%), mp 133–137 °C, ^1^H-NMR (DMSO-*d*_6_, 400 MHz) *δ* 10.09 (s, 1 H), 10.01 (s, 1 H), 9.97 (s, 1 H), 8.67 (d, *J* = 9.2 Hz, 1 H), 7.93–7.84 (m, 3 H), 6.89–6.86 (m, 2 H), 4.01 (s, 3 H), HR-FABMS Calcd for C_14_H_13_FN_3_O_4_S (M^+^ + H): 338.0605, Found: 338.0606.

1-(5-Chloro-2-hydroxyphenyl)-3-(2-methoxy-4-nitrophenyl)thiourea (**1j**)

Yellowish brown solid (86.0%), mp 153–154 °C, ^1^H-NMR (DMSO-*d*_6_, 400 MHz) *δ* 10.27 (s, 1 H), 10.08 (s, 1 H), 9.97 (s, 1 H), 8.69 (d, *J* = 9.2 Hz, 1 H), 8.01 (d, *J* = 2.4 Hz, 1 H), 7.89 (dd, *J* = 9.0, 2.6 Hz, 1 H), 7.84 (d, *J* = 2.4 Hz, 1 H), 7.07 (dd, *J* = 8.6, 2.6 Hz, 1 H), 6.92 (d, *J* = 8.4 Hz, 1 H), 4.01 (s, 3 H), HR-FABMS Calcd for C_14_H_12_ClN_3_O_4_S (M^+^ + H): 354.0315, Found: 354.0319.

1-(2-Hydroxy-5-methylphenyl)-3-(2-methoxy-4-nitrophenyl)thiourea (**1k**)

Yellowish brown solid (94.3%), mp 131–137 °C, ^1^H-NMR (CDCl_3_, 400 MHz) *δ* 8.85 (dd, *J* = 8.8, 4.4 Hz, 1 H), 8.36 (brs, 1 H), 7.94 (dd, *J* = 8.8, 4.4 Hz, 1 H), 7,73 (d, *J* = 2.0 Hz, 1 H), 7.60 (brs, 1 H), 7.13 (d, *J* = 4.8 Hz, 1 H), 6.98 (d, *J* = 8.4 Hz, 1 H), 5.72 (brs, 1 H), 3.86 (s, 3 H), 2.33 (s, 3 H), HR-FABMS Calcd for C_15_H_16_N_3_O_4_S (M^+^ + H): 334.3702, Found: 334.0857.

1-(2-Hydroxy-4-methylphenyl)-3-(2-methoxy-4-nitrophenyl)thiourea (**1l**)

Yellow solid (88.7%), mp 140–142 °C, ^1^H-NMR (DMSO-*d*_6_, 400 MHz) *δ* 9.96 (s, 1 H), 9.75 (s, 2 H), 8.83 (s, 1 H), 7.89–7.86 (m, 1 H), 7.81 (d, *J* = 2.0 Hz, 1 H), 7.47 (s, 1 H), 6.73 (s, 1 H), 6.62 (d, *J* = 8.0 Hz, 1 H), 3.98 (s, 3 H), 2.23 (s, 3 H), HR-FABMS Calcd for C_15_H_16_N_3_O_4_S (M^+^ + H): 334,0856, Found: 334.0856.

1-(5-(*tert*-Butyl)-2-hydroxyphenyl)-3-(2-methoxy-4-nitrophenyl)thiourea (**1m**)

Yellow solid (87.9%), mp 63–65 °C, ^1^H-NMR (DMSO-*d*_6_, 400 MHz) *δ* 10.03 (s, 1 H), 9.68 (s, 2 H), 8.81 (d, *J* = 8.8 Hz, 1 H), 7.88 (dd, *J* = 8.8, 2.4 Hz, 1 H), 7.82 (d, *J* = 2.4 Hz, 1 H), 7.74 (s, 1 H), 7.08 (dd, *J* = 8.8, 2.4 Hz, 1 H), 6.84 (d, *J* = 8.4 Hz, 1 H), 3.89 (s, 3 H), 1.24 (s, 9 H), HR-FABMS Calcd for C_18_H_22_N_3_O_4_S (M^+^ + H): 376.1326, Found: 376.1326.

### 4.4. Synthesis of Substituted-N-(4-nitrophenyl)benzo[D]oxazol-2-amine Derivatives (***2a**–**g***)

First, 5 mL of dried acetonitrile was added to potassium hydroxide (KO_2_, 5 eq.) under N_2_ atmosphere. 1-(Substituted-2-hydroxyphenyl)-3-(4-nitrophenyl)thioureas (**1a**–**g**) (100 mg, 1 eq.) solution in acetonitrile was delivered dropwise to the KO_2_-acetonitrile mixture. The reaction mixture was vigorously stirred at room temperature for 16 h under N_2_ atmosphere. After the reaction, cold water was added to the reaction mixture and extracted with dichloromethane, followed by washing with brine solution. After drying with anhydrous MgSO_4_ and filtration, the organic solvent was removed under reduced pressure to prepare the compounds **2a**–**g**.

*N*-(4-Nitrophenyl)benzo[*d*]oxazol-2-amine (**2a**)

Yellow solid (72.4%), mp 219–220 °C, ^1^H-NMR (CDCl_3_, 400 MHz) *δ* 8.31 (d, *J* = 2.0 Hz, 1H), 8.29 (d, *J* = 2.4 Hz, 1H), 7.83 (dd, *J* = 2.4 Hz, 2H), 7.57 (d, *J* = 7.6 Hz, 1H), 7.41 (d, *J* = 8.0 Hz, 1H), 7.30 (t, *J* = 7.7, 1.2 Hz, 1H), 7.22 (t, *J* = 7.8 Hz, 1H), HR-FABMS Calcd for C_13_H_10_N_3_O_3_ (M^+^ + H): 256.2368, Found: 256.0717.

5-Fluoro-*N*-(4-nitrophenyl)benzo[*d*]oxazol-2-amine (**2b**)

Light yellow solid (63.7%), mp 254–256 °C, ^1^H-NMR (CDCl_3_, 400 MHz) *δ* 8.30 (dt, *J* = 7.0, 2.2 Hz, 2H), 9.82 (dt, *J* = 7.0, 2.2 Hz, 2H), 7.27 (s, 1H), 7.31 (q, *J* = 4.4 Hz, 1H), 6.93 (td, *J* = 9.0, 2.4 Hz, 1H), HR-FABMS Calcd for C_13_H_9_FN_3_O_3_ (M^+^ + H): 274.0622, Found: 274.0624.

4-Methyl-*N*-(4-nitrophenyl)benzo[*d*]oxazol-2-amine (**2c**)

Yellow solid (83.8%), mp 227–228 °C, ^1^H-NMR (CDCl_3_, 400 MHz) *δ* 8.30 (dt, *J* = 9.6, 2.6 Hz, 2H), 7.84 (dt, *J* = 8.4, 5.0 Hz, 2H), 7.23 (t, *J* = 4.6, 4.4 Hz, 1H), 7.11 (d, *J* = 1.2 Hz, 1H), 7.10 (s, 1H), 2.59 (s, 3H), HR-FABMS Calcd for C_14_H_12_N_3_O_3_ (M^+^ + H): 270.2634, Found: 270.0877.

5-Methyl-*N*-(4-nitrophenyl)benzo[*d*]oxazol-2-amine (**2d**)

Light yellow solid (67.4%), mp 256–257 °C, ^1^H-NMR (CDCl_3_, 400 MHz) *δ* 8.29 (dd, *J* = 7.2, 2.0 Hz, 2H), 7.82 (dd, *J* = 6.8, 2.0 Hz, 2H), 7.37 (s, 1H), 7.28 (s, 1H), 7.02 (d, *J* = 8.0 Hz, 1H), 2.45 (s, 3H), HR-FABMS Calcd for C_14_H_12_N_3_O_3_ (M^+^ + H): 270.2634, Found: 270.0879.

6-Methyl-*N*-(4-nitrophenyl)benzo[*d*]oxazol-2-amine (**2e**)

Yellow solid (69.0%), mp 226–228 °C, ^1^H-NMR (CDCl_3_, 400 MHz) *δ* 8.29 (dt, *J* = 9.5, 2.7 Hz, 2H), 7.81 (dt, *J* = 9.8, 2.6 Hz, 2H), 7.44 (d, *J* = 8.0 Hz, 1H), 7.22 (s, 1H), 7.11 (d, *J* = 8.4 Hz, 1H), 2.47 (s, 3H), HR-FABMS Calcd for C_14_H_12_N_3_O_3_ (M^+^ + H): 270.2634, Found: 270.0874.

5-(*tert*-Butyl)-*N*-(4-nitrophenyl)benzo[*d*]oxazol-2-amine (**2f**)

Yellow solid (69.0%), mp 217–218 °C, ^1^H-NMR (CDCl_3_, 400 MHz) *δ* 8.29 (dt, *J* = 9.7, 2.7 Hz, 2H), 7.82 (dt, *J* = 9.7, 2.5 Hz, 2H), 7.62 (d, *J* = 2.0 Hz, 1H), 7.31 (d, *J* = 8.8 Hz, 1H), 7.28 (d, *J* = 1.6 Hz, 1H), 1.38 (s, 9H), HR-FABMS Calcd for C_17_H_18_N_3_O_3_ (M^+^ + H): 312.3431, Found: 312.1343.

5-Methoxy-*N*-(4-nitrophenyl)benzo[*d*]oxazol-2-amine (**2g**)

Yellow powder (52%), mp 217–221 °C, ^1^H-NMR (CDCl_3_, 400 MHz) *δ* 8.31–8.27 (m, 2H), 7.83–7.79 (m, 2H), 7.39 (s, 1H), 7.11 (d, *J* = 2.8 Hz, 1H), 6.79 (dd, *J* = 8.8, 2.4 Hz, 1H), 6.63 (d, *J* = 9.2 Hz, 1H), 3.86 (s, 3H), HR-FABMS Calcd for C_14_H_12_N_3_O_4_ (M^+^ + H): 286.0823, Found: 286.0822.

### 4.5. Synthesis of Substituted-N-(2-methoxy-4-nitrophenyl)benzo[D]oxazol-2-amine Derivatives (***2h**–**m***)

Same procedure as 4.4. Synthesis of substituted-*N*-(4-nitrophenyl)benzo[*d*]oxazol-2-amine derivatives with 1-(substituted-2-hydroxyphenyl)-3-(2-methoxy-4-nitrophenyl)thioureas (**1h**–**m**) (1 eq.).

*N*-(2-Methoxy-4-nitrophenyl)benzo[*d*]oxazol-2-amine (**2h**)

Yellow solid (86.9%), mp 168–169 °C, ^1^H-NMR (CDCl_3_, 400 MHz) *δ* 8.68 (d, *J* = 8.8 Hz, 1H), 8.05 (dd, *J* = 9.2, 2.4 Hz, 1H), 7.93 (brs, 1H), 7.81 (d, *J* = 2.4 Hz, 1H), 7.58 (dd, *J* = 7.6, 0.8 Hz, 1H), 7.40 (d, *J* = 8.4 Hz, 1H), 7.25 (dtd, *J* = 33.2, 7.6, 1.2 Hz, 2H), 4.07 (s, 3H), HR-FABMS Calcd for C_14_H_12_N_3_O_4_ (M^+^ + H): 286.2628, Found: 286.0824.

5-Fluoro-*N*-(2-methoxy-4-nitrophenyl)benzo[*d*]oxazol-2-amine (**2i**)

Yellow solid (70.4%), mp 199–201 °C, ^1^H-NMR (DMSO-*d*_6_, 400 MHz) *δ* 10.54 (s, 1H), 8.57 (d, *J* = 8.8 Hz, 1H), 7.99 (d, *J* = 2.4 Hz, 1H), 7.51 (dd, *J* = 8.8, 4.0 Hz, 1H), 7.35 (d, *J* = 8.8 Hz, 1H), 7.01–6.96 (m, 1H), 3.98 (s, 3H), HR-FABMS Calcd for C_14_H_11_FN_3_O_4_ (M^+^ + H): 304.0728, Found: 304.0728.

5-Chloro-*N*-(2-methoxy-4-nitrophenyl)benzo[*d*]oxazol-2-amine (**2j**)

Yellow solid (85.1%) mp 202–204 °C, ^1^H-NMR (DMSO-*d*_6_, 400 MHz) *δ* 10.59 (s, 1H), 8.59 (d, *J* = 9.2 Hz, 1H), 8.03 (dd, *J* = 9.2, 2.4 Hz, 1H), 7.85 (d, *J* = 2.4 Hz, 1H), 7.62 (d, *J* = 2.0 Hz, 1H), 7.59 (d, *J* = 8.8 Hz, 1H), 7.24 (dd, *J* = 8.4, 2.0 Hz, 1H), 4.01 (s, 3H), HR-FABMS Calcd for C_14_H_10_ClN_3_O_4_ (M + H) ^+^ : 320.0433, Found: 320.0435.

*N*-(2-Methoxy-4-nitrophenyl)-5-methylbenzo[*d*]oxazol-2-amine (**2k**)

Yellow solid (78.1%), mp 163–168 °C, ^1^H-NMR (CDCl_3_, 400 MHz) *δ* 8.66 (d, *J* = 8.8 Hz, 1H), 8.04 (dd, *J* = 8.8, 2.8 Hz, 1H), 7.89 (brs, 1H), 7.80 (d, *J* = 2.4 Hz, 1H), 7.37 (s, 1H), 7.26 (d, *J* = 8.4 Hz, 1H), 7.01 (dd, *J* = 8.4, 0.8 Hz, 1H), 4.06 (s, 3H), 2.45 (s, 3H), HR-FABMS Calcd for C_15_H_14_N_3_O_4_ (M^+^ + H): 300.2894, Found: 300.0982.

*N*-(2-Methoxy-4-nitrophenyl)-6-methylbenzo[*d*]oxazol-2-amine (**2l**)

Yellow solid (61.9%), mp 205–207 °C, ^1^H-NMR (DMSO-*d*_6_, 400 MHz) *δ* 10.33 (s, 1H), 8.62 (d, *J* = 9.2 Hz, 1H), 8.00 (dd, *J* = 9.2, 2.4 Hz, 1H), 7.79 (d, *J* = 2.4 Hz, 1H), 7.39 (d, *J* = 8.0 Hz, 1H), 7.07 (d, *J* = 8.0 Hz, 1H), 3.99 (s, 3H), 2.40 (s, 3H), HR-FABMS Calcd for C_15_H_14_N_3_O_4_ (M^+^ + H): 300.0979, Found: 300.0980.

5-(*tert*-Butyl)-*N*-(2-methoxy-4-nitrophenyl)benzo[*d*]oxazol-2-amine (**2m**)

Yellow solid (62.4%), mp 150–153 °C, ^1^H-NMR (DMSO-*d*_6_, 400 MHz) *δ* 10.35 (s, 1H), 8.64 (d, *J* = 8.8 Hz, 1H), 8.00 (dd, *J* = 9.2, 2.0 Hz, 1H), 7.80 (s, 1H), 7.53 (s, 1H), 7.41 (d, *J* = 7.6 Hz, 1H), 7.22 (d, *J* = 7.2 Hz, 1H), 3.99 (s, 3H), 1.33 (s, 9H), HR-FABMS Calcd for C_18_H_20_N_3_O_4_ (M^+^ + H): 342.1448, Found: 342.1451.

### 4.6. Synthesis of N^1^-(Substitutedbenzo[D]oxazole-2-yl)benzene-1,4-diamine Derivatives (***3a**–**g***)

First, 10 mL of ethanol was added to substituted-*N*-(4-nitrophenyl)benzo[*d*]oxazol-2-amine (**2a**–**g**) (100 mg, 1 eq.) and Tin(II) chloride (12 eq.). The reaction mixture was ultrasonicated for 3 h at room temperature. Potassium hydroxide solution was added to the reaction solution, and the mixture was extracted with ethyl acetate, followed by washing with brine solution. After drying with anhydrous MgSO_4_ and filtration, the organic solvent was removed under reduced pressure to prepare the compounds **3a**–**f**.

*N*^1^-(Benzo[*d*]oxazol-2-yl)benzene-1,4-diamine (**3a**)

Dark gray solid (40.9%), mp 198–201 °C, ^1^H-NMR (DMSO-*d*_6_, 400 MHz) *δ* 10.02 (brs, 1H), 7.39 (d, *J* = 7.6 Hz, 1H), 7.36 (dd, *J* = 6.8, 2.0 Hz, 2H), 7.33 (s, 1H), 7.15 (td, *J* = 7.6, 0.8 Hz, 1H), 7.04 (td, *J* = 7.8, 1.2 Hz, 1H), 6.58 (dt, *J* = 9.4, 2.6 Hz, 2H), 4.84 (s, 2H), HR-FABMS Calcd for C_13_H_12_N_3_O (M^+^ + H): 226.2539, Found: 226.0975.

*N*^1^-(5-Fluorobenzo[*d*]oxazol-2-yl)benzene-1,4-diamine (**3b**)

Dark gray solid (39.3%), mp 187–191 °C, ^1^H-NMR (DMSO-*d*_6_, 400 MHz) *δ* 10.16 (brs, 1H), 7.39 (q, *J* = 4.3 Hz, 1H), 7.33 (dt, *J* = 9.1, 2.5 Hz, 2H), 7.17 (dd, *J* = 9.4, 2.6 Hz, 1H), 6.84 (td, *J* = 9.3, 2.6 Hz, 1H), 6.57 (dt, *J* = 9.0, 2.4 Hz, 2H), 4.87 (s, 2H), HR-FABMS Calcd for C_13_H_11_FN_3_O (M^+^ + H): 244.0881, Found: 244.0885.

*N*^1^-(4-Methylbenzo[*d*]oxazol-2-yl)benzene-1,4-diamine (**3c**)

Brown solid (75.2%), mp 137–138 °C, ^1^H-NMR (DMSO-*d*_6_, 400 MHz) *δ* 9.94 (brs, 1H), 7.36 (dd, *J* = 6.8, 2.0 Hz, 2H), 7.21 (d, *J* = 7.6 Hz, 1H), 6.99–6.91 (m, 2H), 6.59 (d, *J* = 2.0 Hz, 1H), 6.57 (d, *J* = 2.4 Hz, 1H), 4.83 (s, 2H), 2.41 (s, 3H), HR-FABMS Calcd for C_14_H_14_N_3_O (M^+^ + H): 240.2805, Found: 240.1140.

*N*^1^-(5-Methylbenzo[*d*]oxazol-2-yl)benzene-1,4-diamine (**3d**)

Dark gray solid (64.0%), mp 170–178 °C, ^1^H-NMR (DMSO-*d*_6_, 400 MHz) *δ* 9.96 (s, 1H), 7.35 (d, *J* = 2.0 Hz, 1H), 7.33 (d, *J* = 1.6 Hz, 1H), 7.26 (d, *J* = 8.4 Hz, 1H), 7.15 (s, 1H), 6.85 (dd, *J* = 8.0, 0.8 Hz, 1H), 6.57 (dt, *J* = 9.6, 2.8 Hz, 2H), 4.85 (brs, 2H), 2.34 (s, 3H), HR-FABMS Calcd for C_14_H_14_N_3_O (M^+^ + H): 240.2805, Found: 240.1137.

*N*^1^-(6-Methylbenzo[*d*]oxazol-2-yl)benzene-1,4-diamine (**3e**)

Greyish pink solid (49.4%), mp 153–154 °C, ^1^H-NMR (DMSO-*d*_6_, 400 MHz) *δ* 9.94 (brs, 1H), 7.35 (dt, *J* = 9.2, 2.6 Hz, 2H), 7.22 (d, *J* = 3.2 Hz, 1H), 7.20 (s, 1H), 6.97 (dd, *J* = 8.0, 0.8 Hz, 1H), 6.57 (dt, *J* = 9.0, 2.6 Hz, 2H), 4.82 (s, 2H), 2.36 (s, 3H), HR-FABMS Calcd for C_14_H_14_N_3_O (M^+^ + H): 240.2805, Found: 240.1137.

*N*^1^-(5-(*tert*-Butyl)benzo[*d*]oxazol-2-yl)benzene-1,4-diamine (**3f**)

Gray solid (84.4%), mp 185–190 °C, ^1^H-NMR (DMSO-*d*_6_, 400 MHz) *δ* 9.96 (s, 1H), 7.36 (dt, *J* = 9.2, 2.8 Hz, 3H), 7.28 (d, *J* = 8.4 Hz, 1H), 7.07 (dd, *J* = 8.4, 2.0 Hz, 1H), 6.57 (dt, *J* = 9.2, 2.6 Hz, 2H), 4.82 (s, 2H), 1.31 (s, 9H), HR-FABMS Calcd for C_17_H_20_N_3_O (M^+^ + H): 282.3602, Found: 282.1608.

*N*^1^-(5-Methoxybenzo[*d*]oxazol-2-yl)benzene-1,4-diamine (**3g**)

Dark brown solid (51.3%), mp 192–201 °C, ^1^H-NMR (DMSO-*d*_6_, 400 MHz) *δ* 9.99 (brs, 1H), 7.34 (dt, *J* = 9.6, 2.6 Hz, 2H), 7.27 (d, *J* = 8.4 Hz, 1H), 6.93 (d, *J* = 2.4 Hz, 1H), 6.60 (d, *J* = 2.8 Hz, 1H), 6.57 (dt, *J* = 9.2, 2.6 Hz, 2H), 4.83 (s, 2H), 3.75 (s, 3H), HR-FABMS Calcd for C_14_H_14_N_3_O_2_ (M^+^ + H): 256.2799.

### 4.7. Synthesis of 2-Methoxy-N^1^-(substitutedbenzo[D]oxazol-2-Yl)benzene-1,4-diamine Derivatives (***3h**–**m***)

Same procedure as 4.5. Synthesis of *N*^1^-(substitutedbenzo[*d*]oxazole-2-yl)benzene-1,4-diamine derivatives with substituted-*N*-(2-methoxy-4-nitrophenyl)benzo[*d*]oxazol-2-amine (**2h**–**m**) (1 eq.).

*N*^1^-(Benzo[*d*]oxazol-2-yl)-2-methoxybenzene-1,4-diamine (**3h**)

Gray solid (58.2%), mp 110–112 °C, ^1^H-NMR (DMSO-*d*_6_, 400 MHz) *δ* 9.06 (s, 1H), 7.33 (t, *J* = 8.0 Hz, 2H), 7.25 (d, *J* = 7.6 Hz, 1H), 7.14–7.10 (m, 1H), 7.02–6.97 (m, 1H), 6.31 (d, *J* = 2.4 Hz, 1H), 6.17 (dd, *J* = 8.8, 2.4 Hz, 1H), 5.05 (s, 2H), 3.70 (s, 3H), HR-FABMS Calcd for C_14_H_14_N_3_O_2_ (M^+^ + H): 256.1081, Found: 256.1082.

*N*^1^-(5-Fluorobenzo[*d*]oxazol-2-yl)-2-methoxybenzene-1,4-diamine (**3i**)

White solid (41.5%), mp 118–120 °C, ^1^H-NMR (DMSO-*d*_6_, 400 MHz) *δ* 9.25 (s, 1H), 7.34 (dd, *J* = 8.8, 4.8 Hz, 1H), 7.246 (d, *J* = 8.4 Hz, 1H), 7.07 (dd, *J* = 5.6, 2.4 Hz, 1H), 6.82–6.76 (m, 1H), 6.31 (d, *J* = 2.4 Hz, 1H), 6.17 (dd, *J* = 8.4, 2.4 Hz, 1H), 5.09 (s, 2H), 3.70 (s, 3H), HR-FABMS Calcd for C_14_H_13_FN_3_O_2_ (M^+^ + H): 274.0986, Found: 274.0989.

*N*^1^-(5-Chlorobenzo[*d*]oxazol-2-yl)-2-methoxybenzene-1,4-diamine (**3j**)

Gray solid (42.3%), mp 117–120 °C, ^1^H-NMR (DMSO-*d*_6_, 400 MHz) *δ* 9.45 (s, 2H), 9.29 (s, 1H), 7.43 (d, *J* = 8.0 Hz, 2H), 7.32 (d, *J* = 2.0 Hz, 1H), 7.06–7.03 (m, 1H), 6.46 (s, 1H), 6.34 (d, *J* = 8.0 Hz, 1H), 3.73 (s, 3H), HR-FABMS Calcd for C_14_H_13_ClN_3_O_2_ (M^+^ + H): 290.0691, Found: 290.0692.

2-Methoxy-N^1^-(5-methylbenzo[d]oxazol-2-yl)benzene-1,4-diamine (**3k**)

Brown solid (44.4%), mp 123–125 °C, ^1^H-NMR (DMSO-*d*_6_, 400 MHz) *δ* 8,98 (s, 1H), 7.31 (d, *J* = 8.4 Hz, 1H), 7.20 (d, *J* = 8.4 Hz, 1H), 7.05 (s, 1H), 6.80 (d, *J* = 9.2 Hz, 1H), 6.31 (d, *J* = 2.4 Hz, 1H), 6.17 (dd, *J* = 8.4, 2.4 Hz, 1H), 5.05 (s, 2H), 3.70 (s, 3H), 2.32 (s, 3H), HR-FABMS Calcd for C_15_H_16_N_3_O_2_ (M^+^ + H): 270.1237, Found: 270.1238.

2-Methoxy-*N*^1^-(6-methylbenzo[*d*]oxazol-2-yl)benzene-1,4-diamine (**3l**)

Gray solid (57.0%), mp 186–189 °C, ^1^H-NMR (DMSO-*d*_6_, 400 MHz) *δ* 8.94 (s, 1H), 7.34 (d, *J* = 8.8 Hz, 1H), 7.12 (d, *J* = 8.0 Hz, 1H), 6.93 (dd, *J* = 7.6, 0.8 Hz, 1H), 6.31 (d, *J* = 2.4 Hz, 1H), 6.17 (dd, *J* = 8.0, 2.4 Hz, 1H), 5.03 (s, 2H), 3.70 (s, 3H), 2.34 (s, 3H), HR-FABMS Calcd for C_15_H_16_N_3_O_2_ (M^+^ + H): 270.1237, Found: 270.1240.

*N*^1^-(5-(*tert*-Butyl)benzo[*d*]oxazol-2-yl)-2-methoxybenzene-1,4-diamine (**3m**)

Brown solid (68.8%), mp 185–188 °C, ^1^H-NMR (DMSO-*d*_6_, 400 MHz) *δ* 8.98 (s, 1H), 7.35 (d, *J* = 8.0 Hz, 1H), 7.28 (d, *J* = 1.6 Hz, 1H), 7.23 (d, *J* = 8.4 Hz, 1H), 7.03 (dd, *J* = 8.8, 1.6 Hz, 1H), 6.31 (d, *J* = 2.4 Hz, 1H), 6.17 (dd, *J* = 8.4, 2.4 Hz, 1H), 5.04 (s, 2H), 3.70 (s, 3H), 1.29 (s, 9H), HR-FABMS Calcd for C_18_H_22_N_3_O_2_ (M^+^ + H): 312.1707, Found: 312.1707.

### 4.8. Synthesis of Tert-Butyl (4-((4-(Substitutedbenzo[D]Oxazol-2-ylamino)phenyl)amino)-4-oxobutyl)carbamate (***4a**–**g***)

First, 10 mL of dimethylformamide was added to *N*^1^-(substitutedbenzo[*d*]oxazole-2-yl)benzene-1,4-diamine derivatives (**3a**–**g**) (100 mg, 1 eq.), Boc-GABA-OH (1 eq.) and PyBOP (1.2 eq.) and stirred at 0 °C. Diisopropylethylamine (2 eq.) to the reaction mixture and the reaction mixture was stirred for 16 h at room temperature. After the reaction, 10% HCl solution was added to the reaction mixture and extracted with ethyl acetate, followed by washing with sodium bicarbonate solution and brine solution. After drying with anhydrous MgSO_4_ and filtration, the organic solvent was removed under reduced pressure to prepare the compounds **4a**–**g**.

*Tert*-Butyl (4-((4-((5-fluorobenzo[*d*]oxazol-2-yl)amino)phenyl)amino)-4-oxobutyl)carbamate (**4a**)

Green solid (75.7%), mp 185–180 °C, ^1^H-NMR (DMSO-*d*_6_, 400 MHz) *δ* 10.62 (s, 1H), 9.84 (s, 1H), 7.63 (d, *J* = 8.8 Hz, 2H), 7.58 (d, *J* = 9.2 Hz, 2H), 7.47 (dd, *J* = 8.8, 4.4 Hz, 1H), 7.27 (dd, *J* = 9.2, 2.4 Hz, 1H), 6.94–6.89 (m, 1H), 6.83 (s, 1H), 2.96 (d, *J* = 6.4 Hz, 2H), 2.28 (t, *J* = 7.2 Hz, 2H), 1.69 (t, *J* = 7.2 Hz, 2H), 1.38 (s, 9H), HR-FABMS Calcd for C_22_H_26_FN_4_O_4_ (M^+^ + H): 429.1933, Found: 429.1931.

*Tert*-Butyl (4-((4-(benzo[*d*]oxazol-2-ylamino)phenyl)amino)-4-oxobutyl)carbamate (**4b**)

Light yellow solid (38.1%), mp 124–126 °C, ^1^H-NMR (DMSO-*d*_6_, 400 MHz) *δ* 10.50 (s, 1H), 9.83 (s, 1H), 7.65 (d, *J* = 4.4 Hz, 2H), 7.57 (d, *J* = 9.2 Hz, 2H), 7.47–7.41 (m, 2H), 7.23–7.19 (m, 1H), 7.13–7.09 (m, 1H), 6.83 (s, 1H), 2.95 (t, *J* = 6.4 Hz, 2H), 2.28 (t, *J* = 7.6 Hz, 2H), 1.69 (t, *J* = 7.2 Hz, 2H), 1.38 (s, 9H), HR-FABMS Calcd for C_22_H_27_N_4_O_4_ (M^+^ + H): 411.2027, Found: 411.2025.

*Tert*-Butyl (4-((4-((4-methylbenzo[*d*]oxazol-2-yl)amino)phenyl)amino)-4-oxobutyl)carbamate (**4c**)

Gary solid (33.9%), mp 127–129 °C, ^1^H-NMR (DMSO-*d*_6_, 400 MHz) *δ* 10.43 (s, 1H), 9.83 (s, 1H), 7.67 (d, *J* = 8.8 Hz, 2H), 7.57 (d, *J* = 9.2 Hz, 2H), 7.27 (d, *J* = 7.2 Hz, 1H), 7.04–6.98 (m, 2H), 6.83 (s, 1H), 2.95 (t, *J* = 6.4 Hz, 2H), 2.46 (s, 3H), 2.28 (t, *J* = 7.6 Hz, 2H), 1.75–1.67 (m, 2H), 1.38 (s, 9H), HR-FABMS Calcd for C_23_H_29_N_4_O_4_ (M ^+^ +H): 425.2183, Found: 425.2190.

*Tert*-Butyl (4-((4-((5-methylbenzo[*d*]oxazol-2-yl)amino)phenyl)amino)-4-oxobutyl)carbamate (**4d**)

White powder (34.5%), mp 186–189 ℃, ^1^H-NMR (DMSO-*d*_6_, 400 MHz) *δ* 10.43 (s, 1H), 9.82 (s, 1H), 7.63 (d, *J* = 9.2 Hz, 2H), 7.56 (d, *J* = 9.2 Hz, 2H), 7.32 (d, *J* = 8.4 Hz, 1H), 7.23 (s, 1H), 6.91 (d, *J* = 8.4 Hz, 1H), 6.83 (s, 1H), 2.95 (t, *J* = 7.2 Hz, 2H), 2.36 (s, 3H), 2.27 (t, *J* = 7.6 Hz, 2H), 1.69 (t, *J* = 7.2 Hz, 2H), 1.38 (s, 9H), HR-FABMS Calcd for C_23_H_29_N_4_O_4_ (M ^+^ +H): 425.2183, Found: 425.2184.

*Tert-Butyl (4-((4-((6-methylbenzo[d]oxazol-2-yl)amino)phenyl)amino)-4-oxobutyl)carbamate* (**4e**)

Light brown solid (50.9%), mp 173–176 °C, ^1^H-NMR (DMSO-*d*_6_, 400 MHz) *δ* 10.39 (s, 1H), 9.79 (s, 1H), 7.65–7.60 (m, 2H), 7.55–7.52 (m, 2H), 7.26 (t, *J* = 7.6 Hz, 2H), 6.99–6.96 (m, 1H), 6.80 (s, 1H), 2.92 (t, *J* = 5.6 Hz, 2H), 2.35 (s, 3H), 2.24 (t, *J* = 7.6 Hz, 2H), 1.65 (d, *J* = 7.2 Hz, 2H), 1.35 (s, 9H), HR-FABMS Calcd for C_23_H_29_N_4_O_4_ (M ^+^ +H): 425.2183, Found: 425.2184.

*Tert*-Butyl (4-((4-((5-(*tert*-butyl)benzo[*d*]oxazol-2-yl)amino)phenyl)amino)-4-oxobutyl)-carbamate (**4f**)

Brown oil (60.2%), ^1^H-NMR (DMSO-*d*_6_, 400 MHz) *δ* 10.43 (s, 1H), 9.82 (s, 1H), 7.65 (d, *J* = 9.2 Hz, 2H), 7.56 (d, *J* = 8.8 Hz, 2H), 7.45 (s, 1H), 7.4 (d, *J* = 8.4 Hz, 1H), 7.14 (d, *J* = 6.8 Hz, 1H), 6.83 (s, 1H), 2.96 (d, *J* = 6.4 Hz, 2H), 2.27 (t, *J* = 6.8 Hz, 2H), 1.68 (d, *J* = 7.2 Hz, 2H), 1.38 (s, 9H), 1.32 (s, 9H), HR-FABMS Calcd for C_26_H_35_N_4_O_4_ (M ^+^ +H): 467.2653, Found: 467.2656.

*Tert*-Butyl (4-((4-((5-methoxybenzo[*d*]oxazol-2-yl)amino)phenyl)amino)-4-oxobutyl)carbamate (**4g**)

Ivory solid (90.0%), mp 190–192 °C, ^1^H-NMR (DMSO-*d*_6_, 400 MHz) *δ* 10.45 (s, 1H), 9.82 (s, 1H), 7.63 (d, *J* = 8.8 Hz, 2H), 7.56 (d, *J* = 9.2 Hz, 2H), 7.34 (d, *J* = 9.2 Hz, 1H), 7.02 (d, *J* = 2.4 Hz, 1H), 6.83 (t, *J* = 4.8 Hz, 1H), 6.66 (dd, *J* = 8.4, 2.8 Hz, 1H), 3.77 (s, 3H), 2.96 (dd, *J* = 13.2, 6.8 Hz, 2H), 2.27 (t, *J* = 7.6 Hz, 2H), 1.69 (t, *J* = 7.2 Hz, 2H), 1.38 (s, 9H), HR-FABMS Calcd for C_23_H_29_N_4_O_5_ (M ^+^ +H): 441.2132, Found: 441.2128.

### 4.9. Synthesis of Tert-Butyl (4-((4-(Substitutedbenzo[d]Oxazol-2-ylamino)-3-methoxyphenyl)amino)-4-oxobutyl)carbamate (***4h**–**m***)

Same procedure as 4.8. Synthesis of *tert*-butyl (4-((4-(substitutedbenzo[*d*]oxazol-2-ylamino)phenyl)amino)-4-oxobutyl)carbamate with 2-methoxy-*N*^1^-(substitutedbenzo[*d*]oxazol-2-yl)benzene-1,4-diamine derivatives (**3h**–**m**) (1 eq.).

*Tert*-Butyl (4-((4-(benzo[*d*]oxazol-2-ylamino)-3-methoxyphenyl)amino)-4-oxobutyl)carbamate (**4h**)

Brown oil (81.1%), mp 110–112 °C, ^1^H-NMR (DMSO-*d*_6_, 400 MHz) *δ* 9.90 (s, 1H), 9.50 (s, 1H), 7.93 (d, *J* = 8.4 Hz, 1H), 7.51 (d, *J* = 2.0 Hz, 1H), 7.43–7.35 (m, 1H), 7.19 (dd, *J* = 7.6, 0.8 Hz, 1H), 7.13 (dd, *J* = 8.8, 2.0 Hz, 1H), 7.07 (dd, *J* = 8.0, 1.2 Hz, 1H), 6.84 (s, 1H), 3.80 (s, 3H), 2.97 (d, *J* = 6.4 Hz, 2H), 2.29 (s, 2H), 1.69 (s, 2H), 1.38 (s, 9H), HR-FABMS Calcd for C_23_H_29_N_4_O_5_ (M ^+^ +H): 441.2132, Found: 441.2137.

*Tert*-Butyl (4-((4-((5-fluorobenzo[*d*]oxazol-2-yl)amino)-3-methoxyphenyl)amino)-4-oxobutyl)-carbamate (**4i**)

Light brown solid (59.5%), mp 166–170 °C, ^1^H-NMR (DMSO-*d*_6_, 400 MHz) *δ* 9.91 (s, 1H), 9.67 (s, 1H), 7.84 (d, *J* = 8.4 Hz, 1H), 7.51 (d, *J* = 2.0 Hz, 1H), 7.42 (dd, *J* = 8.8, 4.4 Hz, 1H), 7.19 (dd, *J* = 9.2, 2.8 Hz, 1H), 7.13 (dd, *J* = 8.8, 2.0 Hz, 1H), 6.90–6.84 (m, 2H), 3.80 (s, 3H), 2.96 (t, *J* = 6.8 Hz, 2H), 2.29 (t, *J* = 7.6 Hz, 2H), 1.68 (d, *J* = 6.8 Hz, 2H), 1.38 (s, 9H), HR-FABMS Calcd for C_24_H_31_N_4_O_5_ (M ^+^ +H): 455.2289, Found: 455.2292.

*Tert*-Butyl (4-((4-((5-chlorobenzo[*d*]oxazol-2-yl)amino)-3-methoxyphenyl)amino)-4-oxobutyl)-carbamate (**4j**)

Purple solid (85.4%), mp 60–63 °C, ^1^H-NMR (DMSO-*d*_6_, 400 MHz) *δ* 9.92 (s, 1H), 9.73 (s, 1H), 7.94–7.81 (m, 1H), 7.51 (d, *J* = 2.4 Hz, 1H), 7.45–7.40 (m, 1H), 7.20–7.08 (m, 2H), 6.84 (s, 1H), 3.79 (s, 3H), 2.96 (t, *J* = 6.0 Hz, 2H), 2.29 (t, *J* = 7.6 Hz, 2H), 1.68 (d, *J* = 7.2 Hz, 2H), 1.38 (s, 9H), HR-FABMS Calcd for C_23_H_28_ClN_4_O_5_ (M ^+^ +H): 475.1743, Found: 475.1743.

*Tert*-Butyl (4-((3-methoxy-4-((5-methylbenzo[*d*]oxazol-2-yl)amino)phenyl)amino)-4-oxobutyl)-carbamate (**4k**)

White solid (73.9%), mp 88–85 °C, ^1^H-NMR (DMSO-*d*_6_, 400 MHz) *δ* 9.90 (s, 1H), 9.43 (s, 1H), 7.92 (d, *J* = 8.8 Hz, 1H), 7.50 (d, *J* = 2.0 Hz, 1H), 7.28 (d, *J* = 7.6 Hz, 1H), 7.12 (dd, *J* = 8.8, 2.0 Hz, 1H), 6.83 (d, *J* = 5.6 Hz, 1H), 3.80 (s, 3H), 3.31–2.99 (m, 2H), 2.35 (s, 3H), 2.29 (t, *J* = 7.6 Hz, 2H), 1.69 (t, *J* = 6.8 Hz, 2H), 1.38 (s, 9H), HR-FABMS Calcd for C_24_H_31_N_4_O_5_ (M ^+^ +H): 455.2289, Found: 455.2289.

*Tert*-Butyl (4-((3-methoxy-4-((6-methylbenzo[*d*]oxazol-2-yl)amino)phenyl)amino)-4-oxobutyl)-carbamate (**4l**)

White solid (91.3%), mp 150–153 °C, ^1^H-NMR (DMSO-*d*_6_, 400 MHz) *δ* 9.89 (s, 1H), 9.39 (s, 1H), 7.95 (d, *J* = 8.4 Hz, 1H), 7.50 (d, *J* = 2.0 Hz, 1H), 7.24 (d, *J* = 8.4 Hz, 2H), 7.12 (dd, *J* = 8.8, 2.4 Hz, 1H), 6.99 (d, *J* = 6.8 Hz, 1H), 6.84 (s, 1H), 3.80 (s, 3H), 2.96 (t, *J* = 6.4 Hz, 2H), 2.37 (s, 3H), 2.29 (t, *J* = 7.6 Hz, 2H), 1.69 (t, *J* = 7.6 Hz, 2H), 1.38 (s, 9H), HR-FABMS Calcd for C_24_H_31_N_4_O_5_ (M ^+^ +H): 455.2289, Found: 455.2292.

*Tert*-Butyl (4-((4-((5-(*tert*-butyl)benzo[*d*]oxazol-2-yl)amino)-3-methoxyphenyl)amino)-4-oxo-butyl)carbamate (**4m**)

White solid (83.7%), mp 168–171 °C, ^1^H-NMR (DMSO-*d*_6_, 400 MHz) *δ* 9.89 (s, 1H), 9.43 (s, 1H), 7.96 (d, *J* = 8.4 Hz, 1H), 7.49 (d, *J* = 2.4 Hz, 1H), 7.39 (d, *J* = 1.6 Hz, 1H), 7.31 (d, *J* = 4.4 Hz, 1H), 7.14–7.09 (m, 2H), 6.84 (s, 1H), 3.80 (s, 3H), 2.99–2.94 (m, 2H), 2.29 (t, *J* = 7.2 Hz, 2H), 1.69 (t, *J* = 7.6 Hz, 2H), 1.382 (s, 9H), HR-FABMS Calcd for C_27_H_37_N_4_O_5_ (M ^+^ +H): 497.2758, Found: 497.2762.

### 4.10. Synthesis of 4-Amino-N-(4-(substitutedbenzo[D]oxazol-2-ylamino)phenyl)butanamide (***5a**–**g***)

First, 4 M HCl-dioxane solution (3 mL) was added to the chloroform solution of *tert*-butyl (4-((4-(substitutedbenzo[*d*]oxazol-2-ylamino)phenyl)amino)-4-oxobutyl)carbamate (**4a**–**g**) (100 mg, 1 eq.). The reaction mixture was stirred for 3 h at room temperature. After the reaction, the organic solvent was removed under reduced pressure to prepare the compounds **5a**–**g**.

4-Amino-*N*-(4-(benzo[*d*]oxazol-2-ylamino)phenyl)butanamide (**5a**)

Light yellow solid (90.5%), mp 110–112 °C, ^1^H-NMR (DMSO-*d*_6_, 400 MHz) *δ* 10.54 (s, 1H), 9.99 (s, 1H), 7.80 (s, 2H), 7.67 (d, *J* = 8.8 Hz, 2H), 7.58 (d, *J* = 8.8 Hz, 2H), 7.47 (d, *J* = 7.6 Hz, 1H), 7.42 (d, *J* = 7.2 Hz, 1H), 7.23–7.19 (m, 1H), 7.13–7.09 (m, 1H), 2.87–2.82 (m, 2H), 2.42 (t, *J* = 7.2 Hz, 2H), 1.86 (t, *J* = 7.6 Hz, 2H), HR-FABMS Calcd for C_17_H_19_N_4_O_2_ (M ^+^ +H): 311.1503, Found: 311.1505.

4-Amino-*N*-(4-((5-fluorobenzo[*d*]oxazol-2-yl)amino)phenyl)butanamide (**5b**)

Green solid (69.9%), mp 115–118 °C, ^1^H-NMR (DMSO-*d*_6_, 400 MHz) *δ* 10.66 (s, 1H), 9.98 (s, 1H), 7.75 (s, 2H), 7.66–7.64 (m, 2H), 7.59–7.57 (m, 2H), 7.51–7.46 (m, 1H), 7.27 (dd, *J* = 9.2, 2.4 Hz, 1H), 6.95–6.90 (m, 1H), 2.85 (dd, *J* = 14.8, 6.0 Hz, 2H), 2.41 (t, *J* = 7.2 Hz, 2H), 1.85 (t, *J* = 7.6 Hz, 2H), HR-FABMS Calcd for C_17_H_18_FN_4_O_2_ (M ^+^ +H): 329.1408, Found: 329.1413.

4-Amino-*N*-(4-((4-methylbenzo[*d*]oxazol-2-yl)amino)phenyl)butanamide (**5c**)

Brown solid (34.8%), mp 155–158 ℃, ^1^H-NMR (DMSO-*d*_6_, 400 MHz) *δ* 10.47 (s, 1H), 9.98 (s, 1H), 7.79 (s, 2H), 7.68 (d, *J* = 9.2 Hz, 2H), 7.58 (d, *J* = 8.8 Hz, 2H), 7.27 (d, *J* = 7.2 Hz, 1H), 7.04–6.98 (m, 2H), 2.83 (d, *J* = 7.2 Hz, 2H), 2.46–2.40 (m, 2H), 1.88–1.83 (m, 2H), HR-FABMS Calcd for C_18_H_21_N_4_O_2_ (M ^+^ +H): 325.1659, Found: 325.1659.

4-Amino-*N*-(4-((5-methylbenzo[*d*]oxazol-2-yl)amino)phenyl)butanamide (**5d**)

Gray solid (56.5%), mp 158–160℃, ^1^H-NMR (DMSO-*d*_6_, 400 MHz) *δ* 10.48 (s, 1H), 10.00 (s, 1H), 7.83 (s, 2H), 7.65 (d, *J* = 9.2 Hz, 2H), 7.58 (d, *J* = 9.2 Hz, 2H), 7.33 (d, *J* = 8.0 Hz, 1H), 6.91 (dd, *J* = 8.0, 0.8 Hz, 1H), 2.85 (dd, *J* = 14.8, 6.0 Hz, 2H), 2.42 (t, *J* = 7.2 Hz, 2H), 2.37 (s, 3H), 1.86 (t, *J* = 8.0 Hz, 2H), HR-FABMS Calcd for C_18_H_21_N_4_O_2_ (M ^+^ +H): 325.1659, Found: 325.1658.

4-Amino-*N*-(4-((6-methylbenzo[*d*]oxazol-2-yl)amino)phenyl)butanamide (**5e**)

Light brown solid (43.5%), mp 126–129 ℃, ^1^H-NMR (DMSO-*d*_6_, 400 MHz) *δ* 10.46 (s, 1H), 9.99 (s, 1H), 7.82 (s, 2H), 7.67–7.64 (m, 2H), 7.58 (d, *J* = 7.2 Hz, 2H), 7.29 (d, *J* = 8.0 Hz, 2H), 7.03–7.01 (m, 1H), 2.85 (dd, *J* = 14.8, 6.0 Hz, 2H), 2.43–2.38 (m, 5H), 1.86 (t, *J* = 7.6 Hz, 2H), HR-FABMS Calcd for C_18_H_21_N_4_O_2_ (M ^+^ +H): 325.1659, Found: 325.1661.

4-Amino-*N*-(4-((5-(*tert*-butyl)benzo[*d*]oxazol-2-yl)amino)phenyl)butanamide (**5f**)

Brown solid (24.4%), mp 165–168℃, ^1^H-NMR (DMSO-*d*_6_, 400 MHz) *δ* 10.48 (s, 1H), 10.01 (s, 1H), 7.86 (s, 2H), 7.67 (d, *J* = 8.8 Hz, 2H), 7.58 (d, *J* = 9.2 Hz, 2H), 7.44 (d, *J* = 1.6 Hz, 1H), 7.35 (d, *J* = 8.4 Hz, 1H), 7.14 (dd, *J* = 8.0, 1.6 Hz, 1H), 2.83 (s, 2H), 2.42 (s, 2H), 1.88–1.81 (m, 2H), 1.32 (s, 9H), HR-FABMS Calcd for C_21_H_27_N_4_O_2_ (M ^+^ +H): 367.2129, Found: 367.2133.

4-Amino-*N*-(4-((5-methoxybenzo[*d*]oxazol-2-yl)amino)phenyl)butanamide (**5g**)

Ivory solid (84.7%), mp 216–219 ℃, ^1^H-NMR (DMSO-*d*_6_, 400 MHz) *δ* 10.51 (s, 1H), 10.01 (s, 1H), 7.86 (s, 2H), 7.65 (d, *J* = 9.2 Hz, 2H), 7.58 (d, *J* = 8.8 Hz, 2H), 7.35 (d, *J* = 8.8 Hz, 1H), 7.01 (d, *J* = 2.0 Hz, 1H), 6.67 (dd, *J* = 8.4, 2.8 Hz, 1H), 3.77 (s, 3H), 2.87–2.80 (m, 2H), 2.42 (t, *J* = 7.2 Hz, 2H), 1.86 (t, *J* = 8.0 Hz, 2H), HR-FABMS Calcd for C_18_H_21_N_4_O_3_ (M ^+^ +H): 341.1608, Found: 325.1607.

### 4.11. Synthesis of 4-Amino-N-(4-(substitutedbenzo[d]oxazol-2-ylamino)-3-methoxyphenyl)butanamide (***5h**–**m***)

Same procedure as 4.10. Synthesis of 4-amino-*N*-(4-(substitutedbenzo[*d*]oxazol-2-ylamino)phenyl)butanamide with *tert*-butyl (4-((4-(substitutedbenzo[*d*]oxazol-2-ylamino)-3-methoxyphenyl)amino)-4-oxobutyl)carbamate (**5h**–**m**) (1 eq.).

4-Amino-*N*-(4-(benzo[*d*]oxazol-2-ylamino)-3-methoxyphenyl)butanamide (**5h**)

White solid (87.7%), mp 98–100 °C, ^1^H-NMR (DMSO-*d*_6_, 400 MHz) *δ* 10.08 (s, 1H), 9.57 (s, 1H), 7.95 (d, *J* = 8.4 Hz, 1H), 7.83 (s, 2H), 7.50 (d, *J* = 2.4 Hz, 1H), 7.43 (d, *J* = 7.2 Hz, 1H), 7.36 (d, *J* = 7.6 Hz, 1H), 7.21–7.15 (m, 1H), 7.07–7.02 (m, 1H), 3.81 (s, 3H), 2.89–2.83 (m, 2H), 2.44 (t, *J* = 14.4 Hz, 2H), 1.87 (t, *J* = 8.0 Hz, 2H), HR-FABMS Calcd for C_18_H_21_N_4_O_3_ (M ^+^ +H): 341.1608, Found: 341.1609.

4-Amino-*N*-(4-((5-fluorobenzo[*d*]oxazol-2-yl)amino)-3-methoxyphenyl)butanamide (**5i**)

White solid (56.4%), mp 193–195 ℃, ^1^H-NMR (DMSO-*d*_6_, 400 MHz) *δ* 10.10 (s, 1H), 9.70 (s, 1H), 7.86 (d, *J* = 8.4 Hz, 3H), 7.50 (d, *J* = 2.4 Hz, 1H), 7.42 (dd, *J* = 8.0, 3.6 Hz, 1H), 7.21–7.15 (m, 2H), 6.91–6.85 (m, 1H), 3.80 (s, 3H), 2.85 (d, *J* = 8.8 Hz, 2H), 2.44 (t, *J* = 7.2 Hz, 2H), 1.87 (t, *J* = 8.0 Hz, 2H), HR-FABMS Calcd for C_23_H_28_FN_4_O_5_ (M ^+^ +H): 459.2038, Found: 459.2041.

4-Amino-*N*-(4-((5-chlorobenzo[*d*]oxazol-2-yl)amino)-3-methoxyphenyl)butanamide (**5j**)

Purple solid (37.3%), mp 110–113 °C, ^1^H-NMR (DMSO-*d*_6_, 400 MHz) *δ* 10.09 (s, 1H), 9.75 (s, 1H), 7.83 (s, 2H), 7.50 (s, 1H), 7.45 (d, *J* = 8.4 Hz, 1H), 7.19–7.15 (m, 1H), 7.07 (dd, *J* = 8.4, 2.0 Hz, 1H), 3.80 (s, 3H), 2.88–2.82 (m, 2H), 2.44 (t, *J* = 7.6 Hz, 2H), 1.87 (t, *J* = 7.6 Hz, 2H), HR-FABMS Calcd for C_18_H_20_ClN_4_O_3_ (M ^+^ +H): 375.1218, Found:375.1218.

4-Amino-*N*-(3-methoxy-4-((5-methylbenzo[*d*]oxazol-2-yl)amino)phenyl)butanamide (**5k**)

White solid (95.7%), mp 166–168 °C, ^1^H-NMR (DMSO-*d*_6_, 400 MHz) *δ* 10.05 (s, 1H), 9.50 (s, 1H), 7.93 (d, *J* = 8.8 Hz, 1H), 7.79 (s, 2H), 7.49 (d, *J* = 2.4 Hz, 1H), 7.29 (d, *J* = 8.4 Hz, 1H), 7.16 (dd, *J* = 8.4, 2.4 Hz, 2H), 6.89 (dd, *J* = 8.0, 0.8 Hz, 1H), 3.80 (s, 3H), 2.88–2.83 (m, 3H), 2.43 (t, *J* = 7.2 Hz, 2H), 2.35 (s, 3H), 1.87 (t, *J* = 8.0 Hz, 2H), HR-FABMS Calcd for C_19_H_23_N_4_O_3_ (M ^+^ +H): 355.1765, Found:355.1762.

4-Amino-*N*-(3-methoxy-4-((6-methylbenzo[*d*]oxazol-2-yl)amino)phenyl)butanamide (**5l**)

White solid (69.2%), mp 215–217 °C, ^1^H-NMR (DMSO-*d*_6_, 400 MHz) *δ* 10.20 (s, 1H), 9.78 (s, 1H), 8.01 (s, 2H), 7.89 (d, *J* = 8.8 Hz, 1H), 7.53 (d, *J* = 2.0 Hz, 1H), 7.29 (s, 1H), 7.24 (d, *J* = 7.6 Hz, 1H), 7.19 (dd, *J* = 9.2, 2.0 Hz, 1H), 7.02 (d, *J* = 7.2 Hz, 1H), 3.80 (s, 3H), 2.83 (t, *J* = 7.2 Hz, 2H), 2.45 (t, *J* = 7.2 Hz, 2H), 2.37 (s, 3H), 1.88 (t, *J* = 7.2 Hz, 2H), HR-FABMS Calcd for C_19_H_23_N_4_O_3_ (M ^+^ +H): 355.1765, Found: 355.1765.

4-Amino-*N*-(4-((5-(*tert*-butyl)benzo[*d*]oxazol-2-yl)amino)phenyl)butanamide (**5m**)

Brown solid (24.4%), mp 165–168 °C, ^1^H-NMR (DMSO-*d*_6_, 400 MHz) *δ* 10.48 (s, 1H), 10.01 (s, 1H), 7.86 (s, 2H), 7.67 (d, *J* = 8.8 Hz, 2H), 7.58 (d, *J* = 9.2 Hz, 2H), 7.44 (d, *J* = 1.6 Hz, 1H), 7.35 (d, *J* = 8.4 Hz, 1H), 7.14 (dd, *J* = 8.0, 1.6 Hz, 1H), 2.83 (s, 2H), 2.42 (s, 2H), 1.88–1.81 (m, 2H), 1.32 (s, 9H), HR-FABMS Calcd for C_21_H_27_N_4_O_2_ (M ^+^ +H): 367.2129, Found: 367.2133.

### 4.12. Biological Activity Test

#### 4.12.1. Cell Culture

The human keratinocytes HaCaT or the alpha mouse liver 12 (AML-12) cells were obtained from Cell Lines Service GmbH (Eppelheim, Germany). The cells were cultured in Dulbecco’s modified Eagle’s medium (HyClone) supplemented with 10% fetal bovine serum (HyClone) (HaCaT cells) and 1% penicillin/streptomycin (HyClone), and maintained at 37 °C with 5% CO_2_ in a humidified atmosphere. AML-12 cells were pretreated with 10 µM concentrations of compounds 1 h before LPS treatment and followed with LPS (100 ng/mL) for 24 h.

#### 4.12.2. Evaluation of Cell Viability by MTT Assay

Cell viability was measured by MTT assay. HaCaT cells were seeded in 96-well plates at a density of 5.0 × 10^4^ cells/well. On the next day, cells were treated with indicated concentrations of compounds 1 h before LPS treatment. LPS (Sigma-Aldrich) was placed in the growth media for 6 h and then, cells were gently washed twice with growth medium and incubated with 0.5 mg/mL MTT (Sigma-Aldrich) at 37 °C for an hour. The formazan crystals formed by active mitochondria were dissolved in DMSO and A540 for each well was measured with spectrophotometer.

#### 4.12.3. Animals

Ten-week-old male C57BL/6J mice (Jackson Laboratory, Bar Harbor, ME, USA) were used for the experiments. All animal experiments were performed according to protocol approved by the Institutional Animal Care and Use Committee of Ewha Womans University (EWHA IACUC 19–001). The mice were housed in a temperature-controlled room (20–22 °C with a 12 h light:12 h dark cycle. Ten- to twelve-week-old mice were used for the experiments. Compounds were diluted to 200 μL volume (10 mg/kg) with olive oil (vehicle, sigma) injection and injected into the mice (male C57BL/6J, 8 wks) 1 h before LPS administration. LPS (1 mg/kg, 6 h) was resuspended in saline solution and injected i.p. at a dose of 1 mg/kg of body weight. At the end of the trial, mice were sacrificed (Tribromoethanol, 250 mg/kg, i.p.), liver and blood were collected for further analysis.

#### 4.12.4. Measurements of Blood Parameter

Blood was collected from the inferior vena cava and plasma was separated via centrifugation at 3000 rpm for 15 min at 4 °C. Plasma alanine aminotransferase (ALT) and aspartate transaminase (AST) concentrations were determined using the EnzyChrom^TM^ assay kit (BioAssay Systems, Hayward, CA, USA) according to the manufacturer’s recommendation.

#### 4.12.5. Histology and Immunohistology

First, 4% paraformaldehyde-fixed liver tissues were embedded in paraffin using standard procedures. Four-micron thick liver tissue sections were prepared and immunohistochemical staining was performed according to the manufacturer’s instructions. Four-micron thick liver tissue sections were stained with hematoxylin and eosin (H&E) or immunohistochemical characterizations were performed using antibody specific to F4/80 (Abcam, Cambridge, MA, UK). All steps were performed at room temperature, and tissue was rinsed with tap water after each step. Sections were photographed using a Zeiss microscope equipped with AxioCam software (CarlZiess, Thronwood, NY, USA).

#### 4.12.6. Immunoblotting

Protein levels in cells and mouse tissues were evaluated by immunoblot analysis. Cells and tissues were lysed with cold lysis buffer (20 mM HEPES pH 7.0, 0.15 M NaCl, 10% glycerol, 1% Triton X-100, 1 mM EDTA, 1 mM EGTA, 10 mM β-phosphoglycerate, 1 mM Na3VO4, 5 mM NaF, 1 μg/mL aprotinin, 1 μg/mL leupeptin, 100 μM PMSF) using a Polytron Homogenizer or sonicator. The homogenates were centrifuged at 15,000 rpm, 4 °C for 15 min. After the protein concentration of the lysates (supernatants) was quantified using Bradford assay (Bio-Rad, CA, USA), lysates were mixed with sample buffer (62.5 mM Tris-HCl pH 6.8, 10% glycerol, 2% sodium dodecyl sulfate, 0.0125% bromophenol blue, 2.5% β-mercaptoethanol), and heated at 95 °C for 5 min. Samples were loaded onto a sodium dodecyl sulfate-polyacrylamide gel electrophoresis (SDS-PAGE) gel and separated by electrophoresis in SDS buffer (3 g/L Tris, 14.35 g/L glycine, 1 g/L SDS). The proteins were transferred onto an activated polyvinylidene difluoride (PVDF) membrane with 0.45 μm pore size (Millipore, Darmstadt, Germany) by methanol with transfer buffer (3.03 g/L Tris, 14.17 g/L glycine, 20% methanol). The membrane was incubated with 5% bovine serum albumin (BSA) in tween-20 Tris-buffered saline (TTBS) at room temperature for 20 min using a rocker, followed by incubation at 4 °C overnight on a rocker with antibodies (1:2000 dilution). Immune complexes were detected with horseradish peroxidase (HRP) conjugated-secondary antibodies (Bio-Rad, Hercules, CA, USA) and enhanced with chemiluminescence reagents (Ab Frontier, Daejeon, Korea) using the IQ800 (GE Healthcare, Sweden). The abundance of target proteins was quantitated by densitometric analysis of immunoblots. Bradford assay (SpectraMax M2 Microplate Reader, Molecular Device) data were acquired at Fluorescence Core Imaging Center on Ewha Womans University.

#### 4.12.7. Quantitative Real-Time PCR Analysis

Total RNA was isolated from tissue with the use of the TRIzol reagent (Invitrogen) and was processed for reverse transcription (RT) and real-time PCR analysis with an ABI PRISM 7700 system (PE Biosystems). Data were normalized by the amount of GAPDH mRNA. Primers are listed in Table 3.

### 4.13. Statistical Analysis

All quantitative data were analyzed with GraphPad Prism software and represented as mean ± SD. The statistical significance of the data between control and treatment groups was determined using one-way analysis of variance (ANOVA) followed by the Tukey post-hoc test was used to determine differences between experimental groups. A *p*-value < 0.05 was considered statistically significant.

## 5. Conclusions

We synthesized 13 novel compounds with 4-amino-*N*-(4-(benzo[*d*]oxazol-2-ylamino)phenyl)butanamide moiety and evaluated their biological activities. Five compounds (compound **5d**, **5c**, **5f**, **5m**, and synthetic intermediate **4d**) showed potent inhibition activities on IL-1β and IL-6 mRNA expression in vitro. Further, two compounds (**5f** and **4d**) significantly decreased mRNA levels of IL-1β, IL-6, and TNF-α without hepatotoxicity in vivo. From the in vivo and in vitro test results, we confirmed that our synthesized compounds are effective for the suppression of representative inflammatory cytokines.

## Figures and Tables

**Table 1:** Inhibitory effects of 4-amino-*N*-(4-(benzo[*d*]oxazol-2-ylamino)phenyl)butanamide derivatives on mRNA expression levels of IL-6 and IL-1β.

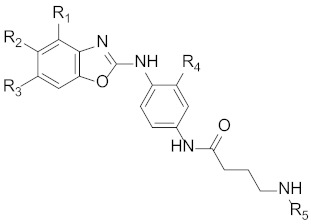							
Compound	R_1_	R_2_	R_3_	R_4_	R_5_	mRNA Expression (%) (10 µM)	
						IL-6	IL-1β
LPS						100.0	
**4d**	H	CH_3_	H	H	Boc	2.40	0.03
**4e**	H	H	CH_3_	H	Boc		
**4l**	H	H	CH_3_	OCH_3_	Boc		
**5a**	H	H	H	H	H		
**5b**	H	F	H	H	H		
**5c**	CH_3_	H	H	H	H	2.08	0.79
**5d**	H	CH_3_	H	H	H	3.39	0.03
**5e**	H	H	CH_3_	H	H		
**5f**	H	C(CH_3_)_3_	H	H	H	3.26	2.83
**5g**	H	OCH_3_	H	H	H		
**5h**	H	H	H	OCH_3_	H		
**5i**	H	F	H	OCH_3_	H		
**5j**	H	Cl	H	OCH_3_	H		
**5k**	H	CH_3_	H	OCH_3_	H		
**5l**	H	H	CH_3_	OCH_3_	H		
**5m**	H	C(CH_3_)_3_	H	OCH_3_	H	4.07	0.49

**Table 2:** IC_50_ (uM) values of the compounds **4d**, **5c**, **5d**, **5f**, and **5m** on suppression of IL-6 mRNA expression.

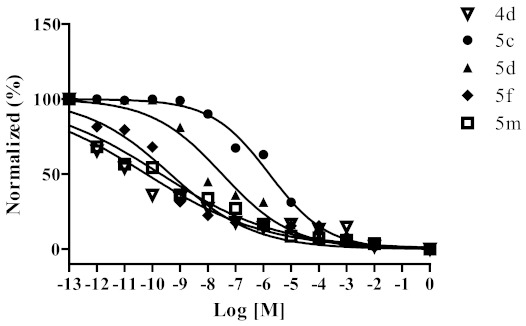		
Compound	Log [M]	IC_50_ (uM)
**4d**	−10.22	6.04 × 10^−5^
**5c**	−5.78	1.64
**5d**	−7.48	3.27 × 10^−2^
**5f**	−9.35	4.44 × 10^−4^
**5m**	−9.70	1.99 × 10^−4^

**Table 3:** Primer sequences of target genes for RT-PCR.

Genes	Forward Primer	Reverse Primer
GAPDH	AGAACATCATCCCTGCATCC	GGTCCTCAGTGTAGCCCAAG
IL-1β	TCGTGCTGTCGGACCCATAT	GTCGTTGCTTGGTTCTCCTTGT
IL-6	ACAACCACGGCCTTCCCTACTT	CACGATTTCCCAGAGAACATGTG
TNF-α	GCCACCACGCTCTTCTG	GGTGTGGGTGAGGAGCA

## Data Availability

Not applicable.
